# Long non-coding RNA *RAB11B-AS1* prevents osteosarcoma development and progression via its natural antisense transcript *RAB11B*

**DOI:** 10.18632/oncotarget.24247

**Published:** 2018-01-13

**Authors:** Zhixu Chen, Zezheng Liu, Yang Yang, Zhaoyin Zhu, Ridong Liang, Bin Huang, Di Wu, Lei Yang, Hai Lu, Dadi Jin, Qingchu Li

**Affiliations:** ^1^ Academy of Orthopedics, Guangdong Province, Department of Orthopedics, The Third Affiliated Hospital, Southern Medical University, Guangzhou 510630, China; ^2^ The State Key Lab of Respiratory Disease, The Institute for Chemical Carcinogenesis, Collaborative Innovation Center for Environmental Toxicity, Guangzhou Medical University, Guangzhou 510182, China

**Keywords:** lnc-RAB11B-AS1, osteosarcoma, RAB11B, proliferation, invasion

## Abstract

Long non-coding RNAs (lncRNAs) have been shown to exert essential roles in development and progression of tumors. Here we discovered a novel lncRNA, RAB11B antisense RNA (RAB11B-AS1), which is markedly down-regulated in human osteosarcoma (OS) and associated with OS metastasis and poor prognosis. We find that reduction of RAB11B-AS1 significantly facilitates proliferation, migration and invasiveness and prevents apoptosis of OS cells and results in lower sensitivity to cisplatin in these cells. In contrast, up-regulation of RAB11B-AS1 suppresses the aggressive behaviors of OS cells. Mechanistically, down-regulation of RAB11B-AS1 elevates its sense-cognate gene RAB11B expression at both mRNA and protein levels. RAB11B-AS1 expression correlates negatively with RAB11B expression in OS tissues. Luciferase reporter assay illuminated that RAB11B-AS1 regulates RAB11B expression through antisense pairing. Most importantly, all the effects of RAB11B-AS1 were abrogated by RAB11B down-regulation. Thus our findings revealed that lnc-RAB11B-AS1 prevents osteosarcoma development and progression via inhibiting RAB11B expression, indicating lnc-RAB11B-AS1 as a potential therapeutic target for osteosarcoma.

## INTRODUCTION

Osteosarcoma (OS), the most common primary bone sarcoma, is one of the malignant tumors threating teenagers’ life and health seriously [1, 2]. Because of lacking effective diagnostic methods, 80 percent of the lymph node micrometastasis of osteosarcoma cannot be detected [3]. Most patients are in middle or advanced stage when they were diagnosed and thus had a bad prognosis. Currently, the main treatment strategy of osteosarcoma is surgical resection with adjuvant chemotherapy. 5-year survival rates for patients with non-metastatic disease reported above 60% to 70% [4, 5], while those with metastatic diseases or relapses result in much poorer prognosis [6]. Therefore, it is exigent to develop novel targets for the diagnosis, treatment and prognosis of osteosarcoma.

Currently, the long non-coding RNA (lncRNA), an RNA molecular that is longer than 200 nucleotides and cannot be translated into a protein, attracts more and more attention. LncRNAs involve in the regulation of almost all life activities, while their known prime function is to regulate expression of genes in different levels, such as epigenetic modifications, endonuclear transportation, transcription, post-transcription and so on [7, 8]. Irregular lncRNA expression has been shown to be concerned with many diseases including tumor. Tumor-related lncRNA is almost twice protein-encoding genes that concerned with tumor [9]. Moreover, lncRNA closely associates with the biological behavior of the tumor, including occurrence, development, invasion and metastasis of tumors [10–12]. Due to these characteristics, lncRNA has potential role in early diagnosis, treatment, and prognosis of tumors. When it comes to osteosarcoma, overexpression of lncRNA *HOTTIP* increases chemoresistance of osteosarcoma cell by activating the Wnt/β-catenin pathway [13]. LncRNA *FGFR3-AS1* promotes osteosarcoma growth through regulating its natural antisense transcript *FGFR3* [14]. Moreover, it is reported that lncRNA *SNHG12* promote the osteosarcoma cells proliferation and migration *in vitro* [15]. But in fact, quite few investigations have been executed on the role of lncRNAs in osteosarcoma until recently, and the underlying mechanisms are largely unknown.

Recently, we discovered a novel lncRNA named *RAB11B-AS1* by bioinformatic analysis. *RAB11B-AS1* locates at chromosome 19q13.2 and belongs to Ras family. *RAB* gene is well known for its role in tumor activation and progression. *RAB11* participates in a great many life activities, such as endocytic membrane traffic, autophagy, invasion of cells and so on [16]. The role of *RAB11* has been implied in many types of tumor, including breast cancer, colorectal carcinoma and skin carcinoma [17–19]. However, the biological function of *RAB11B-AS1* in tumor remains unclear.

In the present study, we aimed to investigate the potential role of *RAB11B-AS1* in osteosarcoma and uncover the underlying mechanisms. Our results suggested that *RAB11B-AS1* significantly suppresses proliferation, migration and invasiveness and promotes apoptosis of OS cells and elevates sensitivity of these cells to cisplatin. Mechanistically, aberrant hyper-methylation of the promoter region might be responsible for decreased *RAB11B-AS1* in osteosarcoma and *RAB11B-AS1* exerts its effects in osteosarcoma via its sense-cognate gene *RAB11B*. Thus our study identified lnc-*RAB11B-AS1* as a novel biomarker and therapeutic target for osteosarcoma.

## RESULTS

### Lnc-*RAB11B-AS1* exhibits decreased expression in osteosarcoma tissues, and correlates with poor prognosis

**Figure 1:**
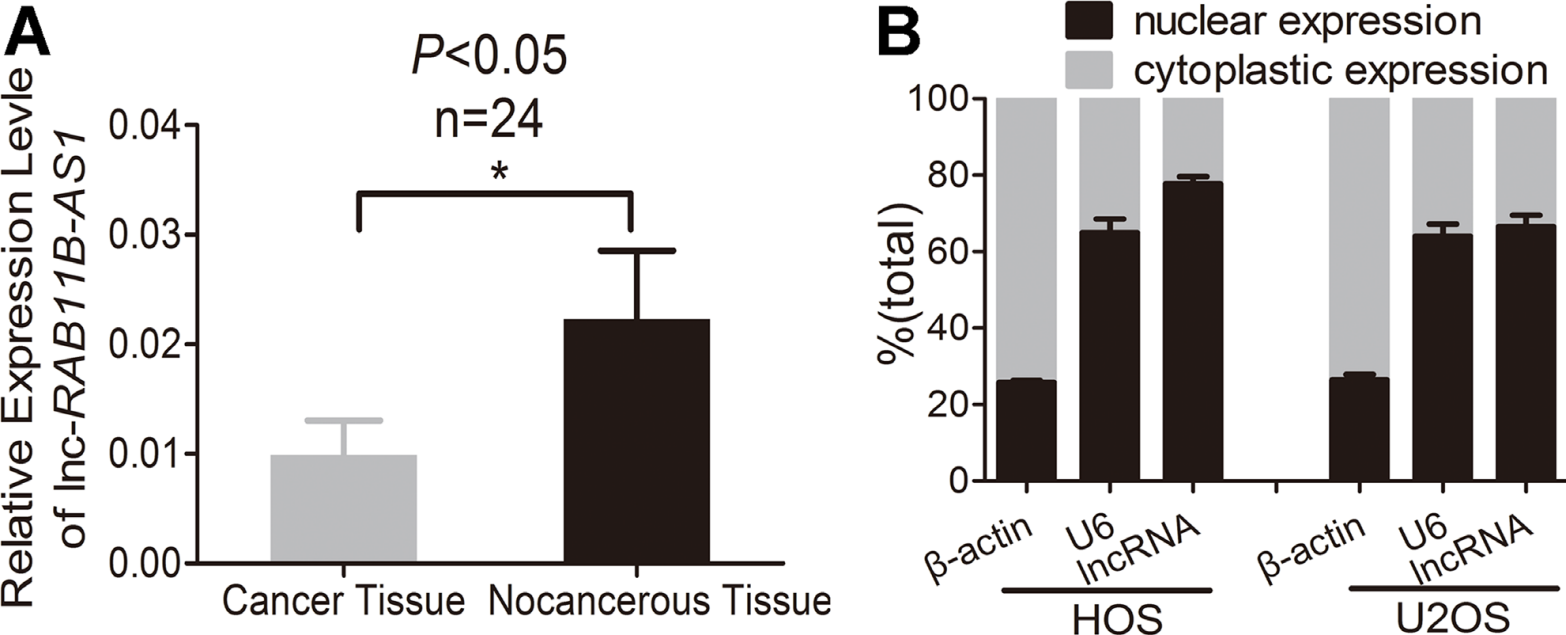
Osteosarcoma tissues exhibits lower lnc-*RAB11B-AS1* expression (**A**) Expression of lnc-*RAB11B-AS1* in osteosarcoma tissues (*n* = 24) compared with the paired non-neoplastic tissues (*n* = 24). (**B**) Subcellular location of lnc-*RAB11B-AS1*. Data was presented as mean ± SD. The results were reproducible in three independent experiments. ^*^P < 0.05.

Firstly, we detected lnc-*RAB11B-AS1* expression in osteosarcoma tissues and paired non-neoplastic tissues from 24 patients. As revealed in Figure 1A, lnc-*RAB11B-AS1* presented a significant decrease in osteosarcoma tissues as compared with their paired non-neoplastic tissues. Next, we examined the correlation between *RAB11B-AS1* expression and clinicopathological characteristics of the 24 osteosarcoma samples. Lnc-*RAB11B-AS1* correlated negatively with clinical stage of tumor, indicating a role of lnc-*RAB11B-AS1* in development and poor prognosis of osteosarcoma (Table 1). We then assessed the subcellular location of the lncRNA. Osteosarcoma cell lines HOS and U2OS were chosen randomly and subjected to nuclear and cytoplasmic fractionation analysis. The majority of lnc-*RAB11B-AS1* was shown to be localized in nucleus other than in cytoplasm of cells, suggesting the lncRNA as a transcriptional regulator in osteosarcoma cells (Figure 1B).

**Table 1:** Correlation between the clinicopathological characteristics and expression of *RAB11B-AS1*

Factors	No of patients (*n* = 24), *n* (%)	*RAB11B-AS1* expression		*P*^a^ value
		Low (*n* = 12), *n* (%)	High (*n* = 12), *n* (%)	
**Age**				
≤ 20	6 (25.0)	2 (33.3)	4 (66.7)	0.640
> 20	18 (75.0)	10 (55.6)	8 (44.4)	
**Gender**				
Male	16 (66.7)	9 (56.2)	7 (43.8)	0.667
Female	8 (33.3)	3 (37.5)	5 (62.5)	
**Location**				
Tibia/femur	20 (83.3)	11 (55.0)	9 (45.0)	0.590
Elsewhere	4 (16.7)	1 (25.0)	3 (75.0)	
**Histological type**				
Osteoblastoma	14 (58.3)	9 (64.3)	5 (35.7)	0.214
Else	10 (41.7)	3 (30.0)	7 (70.0)	
**Differentiated degree**				
High/middle	4 (16.7)	1 (25.0)	3 (75.0)	0.590
Low/undifferentiation	20 (83.3)	11 (55.0)	9 (45.0)	
**TNM**				
T1N0M0	6 (25.0)	4 (66.7)	2 (33.3)	0.640
T2N0M0	18 (75.0)	8 (44.4)	10 (55.6)	
**Clinical stage**				
I	11 (45.8)	2 (18.2)	9 (81.8)	0.012
II	13 (54.2)	10 (76.9)	3 (23.1)	

^a^ Fisher's Exact Test *P* value

The expression of *RAB11B-AS1* is measured by qRT-PCR and the cut-off based on the median of the expression of 24 osteosarcoma tissues and classed as low or high *RAB11B-AS1* expression.

### Lnc-*RAB11B-AS1* abrogates osteosarcoma cells proliferation, migration and invasion and facilitated their apoptosis *in vitro*

**Figure 2:**
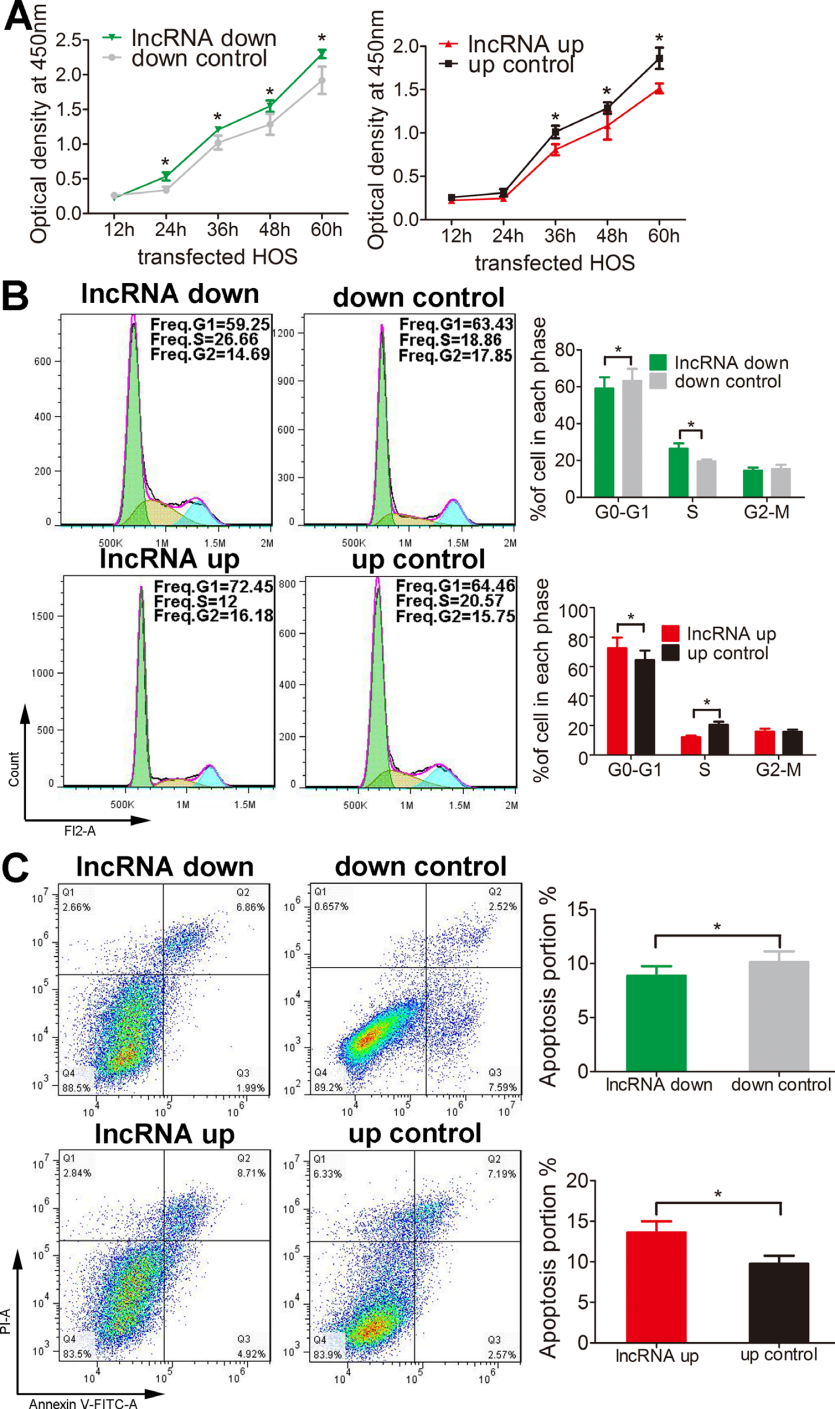
lnc-*RAB11B-AS1* inhibits HOS cells proliferation and promotes HOS cells apoptosis (**A**) Proliferation of HOS cells with up-regulated (up) or down-regulated (down) lnc-RAB11B-AS1 was determined by CCK-8 assay. (**B**) Flow cytometer analysis of the cell cycle distribution of HOS cells with up-regulated or down-regulated lnc-RAB11B-AS1. (**C**) Annexin V-FITC/PI apoptosis assay of HOS cells with up-regulated or down-regulated lnc-*RAB11B-AS1*. Data was presented as mean ± SD. The results were reproducible in three independent experiments. ^*^P < 0.05.

We then examine the role of lnc-*RAB11B-AS1* in osteosarcoma cells. We first tested the proliferative rate of osteosarcoma cells with knockdown or overexpression of lnc-*RAB11B-AS1* using CCK-8 assay. As revealed in Figure 2A and Supplementary Figure 1A, up-regulation of lnc-*RAB11B-AS1* resulted in decreased proliferative rate of osteosarcoma cells, while down-regulation of lnc-*RAB11B-AS1* accelerated proliferation of them. These results suggested that lnc-*RAB11B-AS1* inhibits osteosarcoma cells proliferation. To investigate the underlying mechanism, flow cytometry was then performed in the cells to evaluate cell cycle. HOS cells with reduced lnc-*RAB11B-AS1* showed decreased number in G0-G1 phase and increased number in the S phase, while cells with elevated lnc-*RAB11B-AS1* showed the contrary. These results were copied in U2OS cells (Figure 2B and Supplementary Figure1B), indicating that lnc-*RAB11B-AS1* suppress osteosarcoma cells proliferation by increasing the number of them in G0-G1 phase and reducing the number in the S phase.

**Figure 3:**
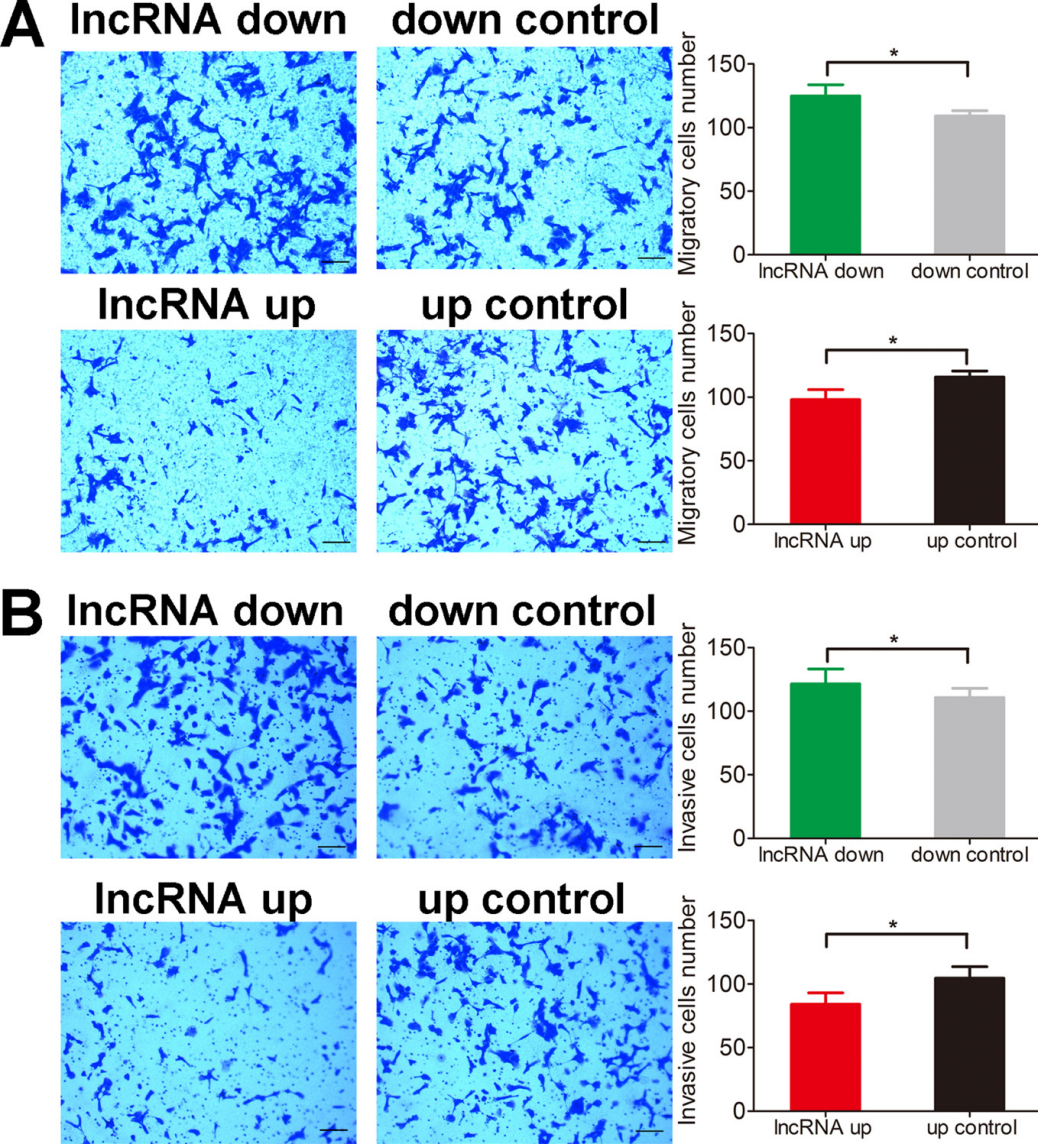
lnc-*RAB11B-AS1* impairs migration and invasion of HOS cells (**A**) Migration assay of HOS cells with up-regulated or down-regulated lnc-*RAB11B-AS1*. (**B**) Transwell invasion assay of HOS cells with up-regulated or down-regulated lnc-*RAB11B-AS1*. Migration and invasion capacities of osteosarcoma cells were measured by transwell chamber assay, and the photographs were randomly selected and taken at × 100 field. Scale bar, 200 μm. Data was presented as mean ± SD. The results were reproducible in three independent experiments. ^*^P < 0.05.

Apoptosis of both HOS and U2OS cells was abrogated by lnc-*RAB11B-AS1* down-regulation and was facilitated by lnc-*RAB11B-AS1* up-regulation (Figure 2C and Supplementary Figure 2), suggesting that lnc-*RAB11B-AS1* promote apoptosis of osteosarcoma cells. In the migration assay, down-regulation of lnc-*RAB11B-AS1* promoted migration of HOS and U2OS cells, while up-regulation of the lncRNA presented contrary effect on the cells (Figure 3A and Supplementary Figure 3A). These results suggested inhibitory role of lnc-*RAB11B-AS1* on osteosarcoma cells migration. Results from invasion assay showed that lnc-*RAB11B-AS1* impair invasion of osteosarcoma cells (Figure 3B and Supplementary Figure 3B).

### Lnc-*RAB11B-AS1* suppresses osteosarcoma growth *in vivo*

**Figure 4:**
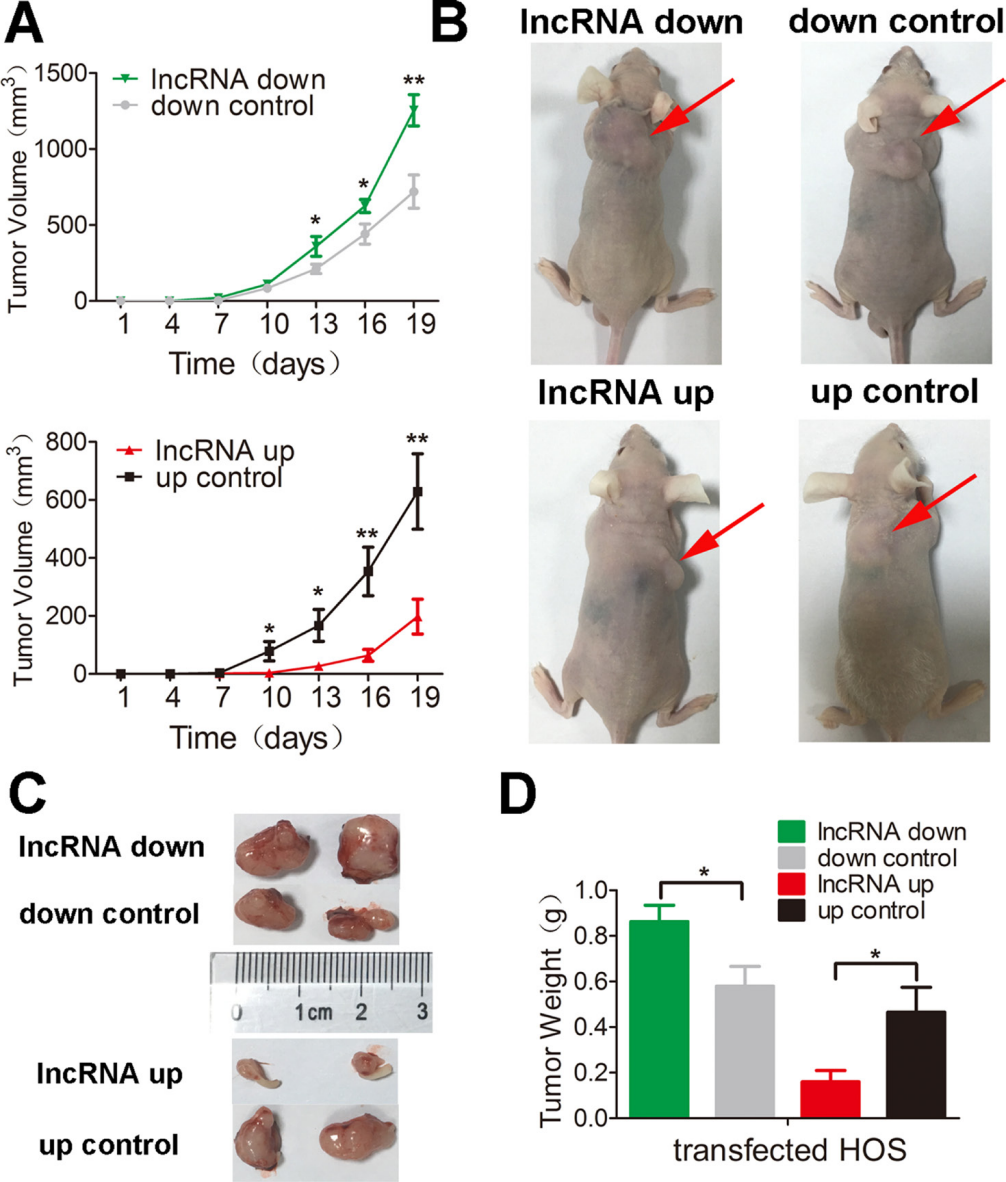
lnc-*RAB11B-AS1* inhibits tumor growth *in vivo* (**A**) Mean volumes of 6 nude mice of each group were calculated every three days after injection of transfected HOS cells. (**B**) Representative photos of mice at 19th day after injection of transfected HOS cells. (**C**) Representative images of xenografted tumors removed from the mice at 19th day after injection. (**D**) Average tumor weights of the mice. Data was presented as mean ± SD. The results were reproducible in three independent experiments. ^*^P < 0.05, ^**^ *P* < 0.01.

**Figure 5:**
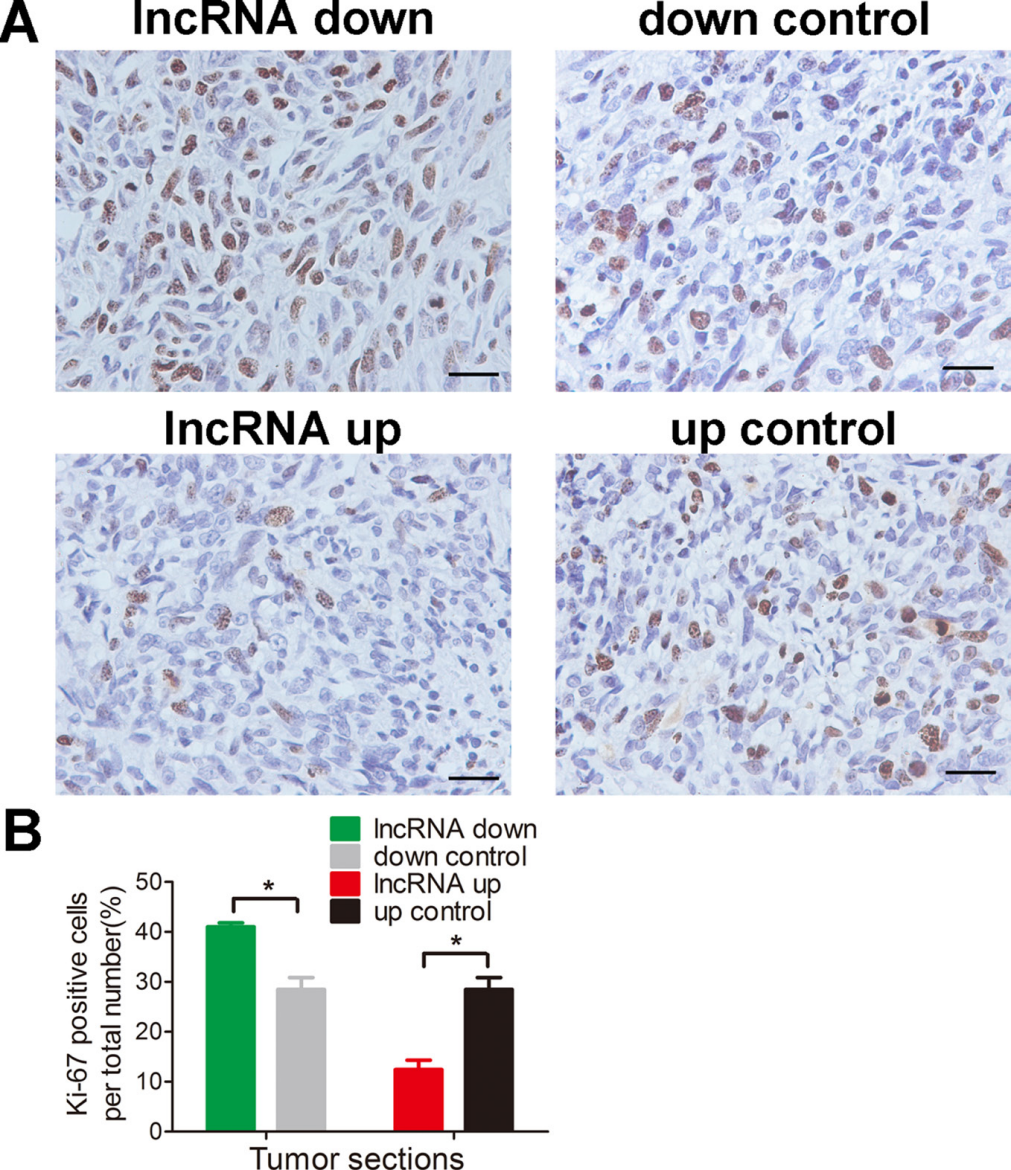
lnc-*RAB11B-AS1* inhibits osteosarcoma cells proliferation *in vivo* (**A**) Immunostaining of Ki-67 in osteosarcoma slices (magnification, × 400; scale bar, 100 μm). (**B**) Quantitative analysis of Ki-67 positive cells over total cells. Data was presented as mean ± SD. The results were reproducible in three independent experiments. ^*^P < 0.05.

**Figure 6:**
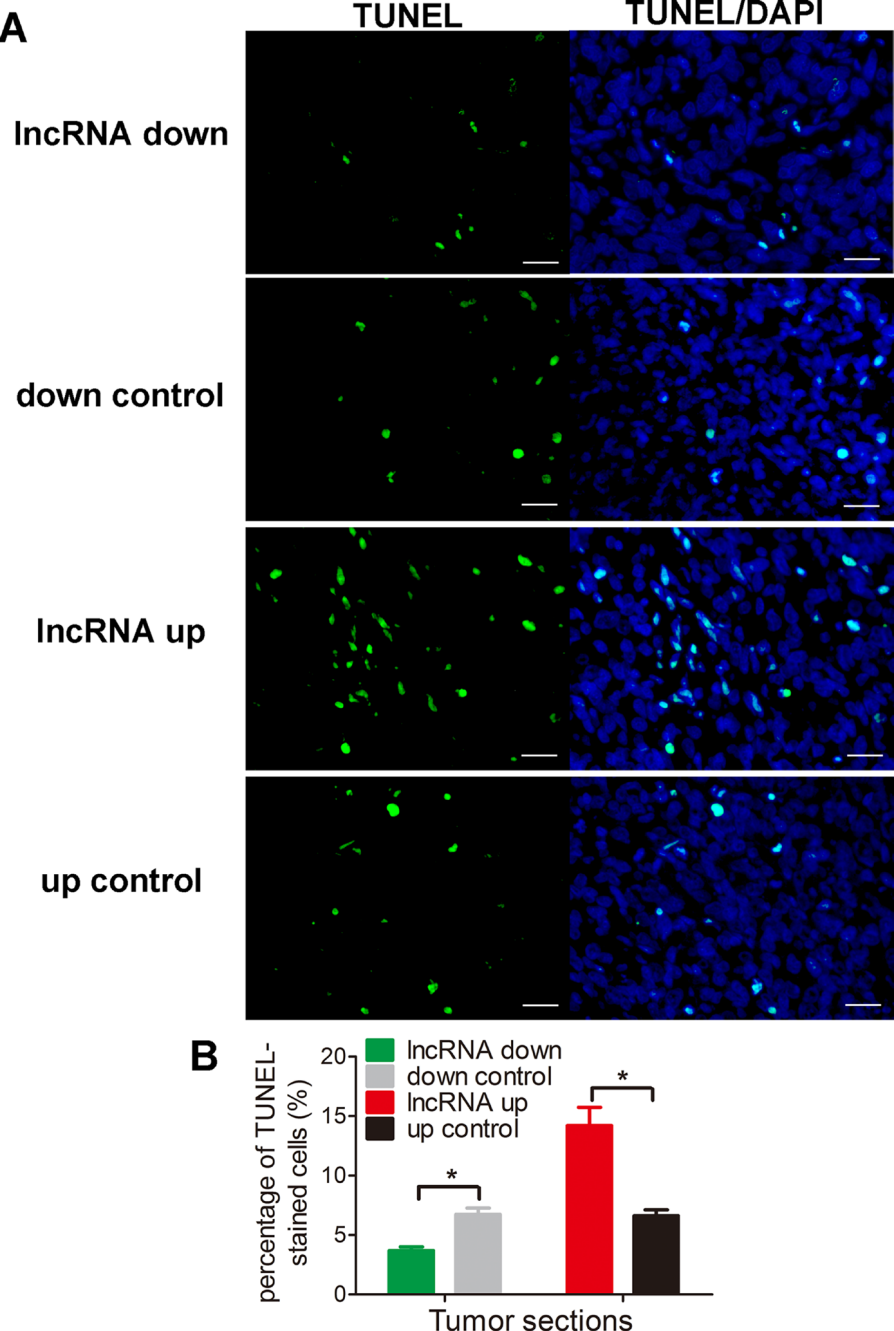
lnc-*RAB11B-AS1* promotes cells apoptosis *in vivo* (**A**) Representative images of TUNEL-stained tumor sections. All the images are 400 ×, scale bar, 100 μm. (**B**) Quantitative analysis of TUNEL-positive cells over total cells. Data was presented as mean ± SD. The results were reproducible in three independent experiments. ^*^P < 0.05.

To test the results presented above *in vivo*, we injected mice with HOS cells that stably expressed either pEZ-Lv206-lnc-*RAB11B-AS1* or psi-LVRH1MP- lnc-*RAB11B-AS1* and monitored tumor growth. HOS cells with overexpressed lnc-*RAB11B-AS1* resulted in slower growth rate of tumor in mice, while those with reduced lnc-*RAB11B-AS1* caused higher growth rate of tumor (Figure 4). To uncover the underlying mechanisms, we performed Ki-67 and TUNEL assay in tumor slices to determine proliferation and apoptosis of osteosarcoma cells *in vivo*. Osteosarcoma cells with decreased lnc-*RAB11B-AS1* showed accelerated proliferation rate, while those with elevated lncRNA exhibited slowed proliferation (Figure 5). Results from TUNEL assay revealed that lnc-*RAB11B-AS1* facilitated apoptosis of osteosarcoma cells *in vivo* (Figure 6). These findings suggest that lnc-*RAB11B-AS1* inhibits osteosarcoma growth *in vivo.*

### Lnc-*RAB11B-AS1* enhances sensitivity of osteosarcoma cells to cisplatin

**Figure 7:**
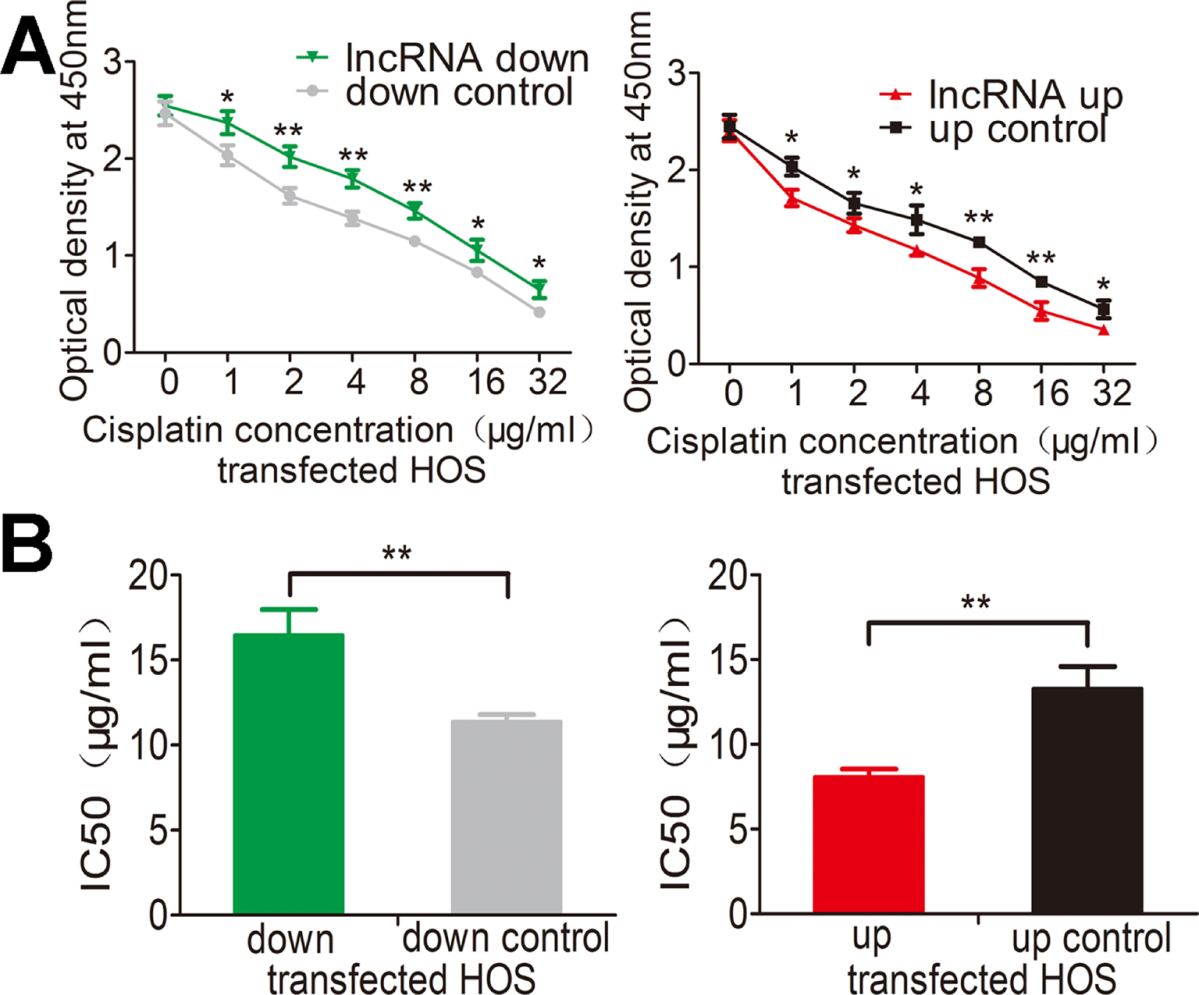
lnc-*RAB11B-AS1* elevates sensitivity of HOS cells to cisplatin (**A**) HOS cell with down-regulated (down) or up-regulated (up) *RAB11B-AS1* were added with increasing concentration of cisplatin and subjected to CCK-8 assay. (**B**) HOS cells with lnc-*RAB11B-AS1* up-regulation were more sensitive to cisplatin (IC50 = 21.18 μg/ml for lnc-*RAB11B-AS1* down-regulation group and IC50 = 10.57 μg/ml for lnc-*RAB11B-AS1* up-regulation group). Data was presented as mean ± SD. The results were reproducible in three independent experiments. ^*^P < 0.05, ^**^P < 0.01.

Genetic differences in tumors influence their response to chemotherapeutic agents and cause inter-individual differences in treatment outcomes. Cisplatin is a common drug used for treating osteosarcoma. We next assessed whether lnc-*RAB11B-AS1* has any role in chemotherapy of osteosarcoma by cisplatin. Treatment with increasing concentrations of cisplatin caused a dose dependent death of osteosarcoma cells. Over-expression of lnc-*RAB11B-AS1* accelerated the death of osteosarcoma cells, while reduction of lnc-*RAB11B-AS1* resulted in resistance of the cells to cisplatin (Figure 7A and Supplementary Figure 4A). Next, we calculated the half maximal inhibitory concentration (IC50) of osteosarcoma cells (Figure 7B and Supplementary Figure 4B), results from which maintained the conclusion that lnc-*RAB11B-AS1* enhanced the sensitivity of osteosarcoma cells to cisplatin.

### Lnc-*RAB11B-AS1* correlates negatively with its sense-cognate gene *RAB11B* in osteosarcoma cells

**Figure 8:**
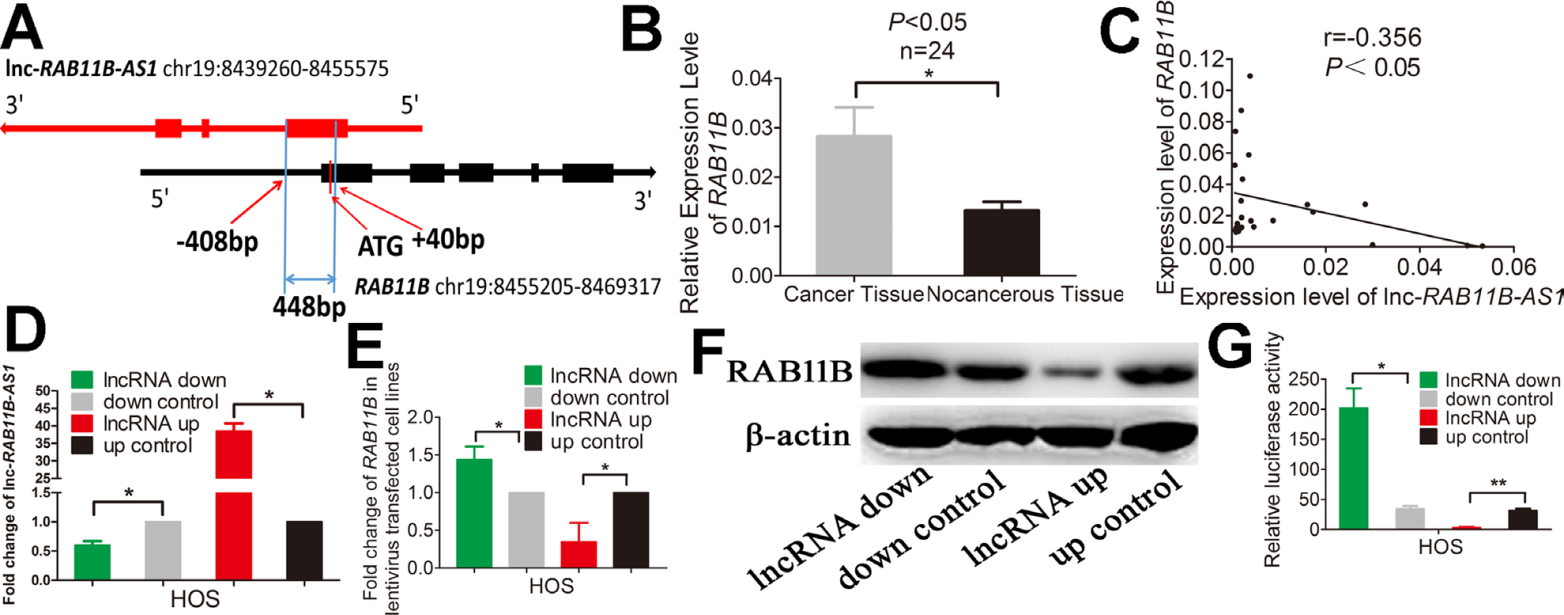
lnc-*RAB11B-AS1* correlates negatively with its sense-cognate gene RAB11B in HOS cells (**A**) Schematic outlining the genomic organization of lnc-*RAB11B-AS1* and *RAB11B*. Arrows show transcription direction and blocks indicate exons. (**B**) qRT-PCR analysis of *RAB11B* in the collected osteosarcoma tissues (*n* = 24) and corresponding non-cancerous tissues (*n* = 24). (**C**) Spearman's correlation analysis showed significant negative correlation between lnc-RAB11B-AS1 and RAB11B expression in osteosarcoma tissues (r=-0.356, *P* < 0.05). (**D**) qRT-PCR analysis of *RAB11B-AS1* in lentivirus transfected HOS cells. (**E**) qRT-PCR analysis of RAB11B in HOS cells with down-regulated or up-regulated RAB11B-AS1. (**F**) Western blot analysis of RAB11B in HOS cells with down-regulated or up-regulated RAB11B-AS1. (**G**)The relative luciferase activity of pGL3-Promoter-RAB11B was markedly increased in the HOS cells with down-regulated lnc-RAB11B-AS1 and was reduced in cells with up-regulated lnc-RAB11B-AS1. Data was presented as mean ± SD. The results were reproducible in three independent experiments. ^*^P < 0.05, ^**^P < 0.01.

We next investigated the mechanisms by which lnc-*RAB11B-AS1* exerts the effects depicted above in osteosarcoma cells. Results from bioinformatic analysis showed that lnc-*RAB11B-AS1* and *RAB11B* formed a ‘head-to-tail’ pairing pattern with 448 nucleotides full complementarity (Figure 8A). Thus we next examined the association between lnc-*RAB11B-AS1* and *RAB11B* mRNA. Expressions of *RAB11B* were detected in the 24 paired tissue samples mentioned above. A significant increase of *RAB11B* level was observed in osteosarcoma tissues as compared with the corresponding non-neoplastic tissues (Figure 8B). Moreover, correlational analysis showed a negative correlation between lnc-*RAB11B-AS1* and *RAB11B* expression in the tissues (Figure 8C). To validate these results *in vitro*, we disrupted lnc-*RAB11B-AS1* expression in osteosarcoma cell lines, and found significant increase of *RAB11B* mRNA in these cells. On the contrary, up-regulation of lnc-*RAB11B-AS1* resulted in reduction of *RAB11B* mRNA in osteosarcoma cells (Figure 8D, Figure 8E, Supplementary Figure 5A and Supplementary Figure 5B). Results from western blotting further confirmed these results at the protein level (Figure 8F and Supplementary Figure 5C). Next, luciferase reporter assay was conducted to explore the interaction between lnc-*RAB11B-AS1* and the *RAB11B*. Reduction of lnc-*RAB11B-AS1* caused increased luciferase activity of pGL3-Promoter-*RAB11B* in osteosarcoma cells, while up-regulation of the lncRNA resulted in contrary effect (Figure 8G and Supplementary Figure 5D). These results demonstrated negative correlation between lnc-*RAB11B-AS1* and *RAB11B* in osteosarcoma cells.

### Lnc-RAB11B-AS1 prevents osteosarcoma progression via down-regulating RAB11B

**Figure 9:**
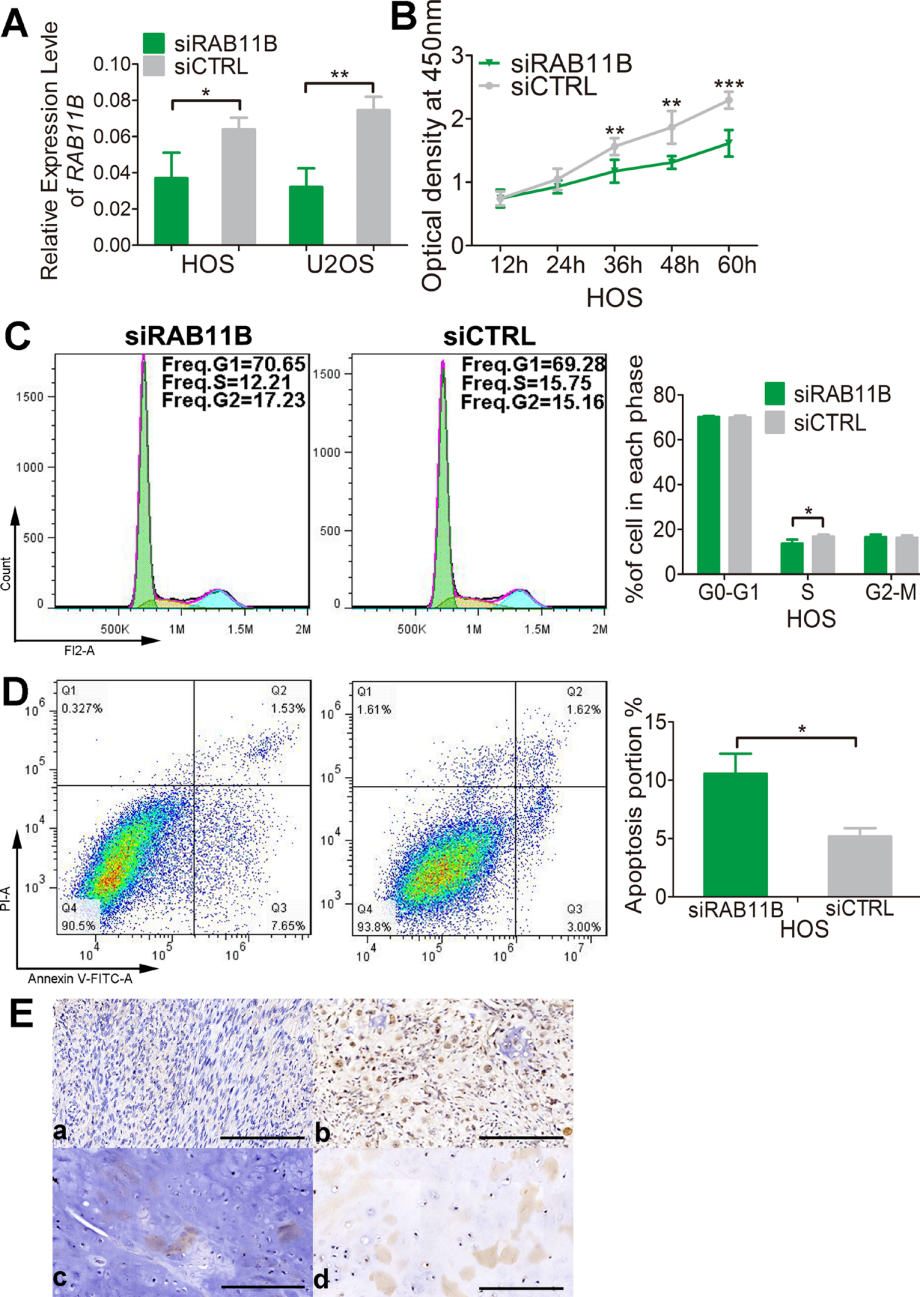
*RAB11B* promotes osteosarcoma progression (**A**) qRT-PCR analysis of RAB11B in HOS cells and U2OS cells with disrupted RAB11B or not. (**B**) Proliferation of HOS cells with disrupted RAB11B or not was determined by CCK-8 assay. (**C**) Flow cytometer analysis of cell cycle distribution in HOS cells with disrupted RAB11B or not. (**D**) HOS cells with disrupted RAB11B or not were subjected to apoptosis analysis. (**E**) Analysis of *RAB11B* expression in osteosarcoma tissues (*n* = 145) by immunochemistry. Scale bar, 100 μm. *RAB11B* positive cells were stained brown. (a, b) showed RAB11B expression in bone tissue and (c, d) presented *RAB11B* expression in cartilage tissue. Data was presented as mean ± SD. The results were reproducible in three independent experiments. ^*^P < 0.05, ^**^P < 0.01, ^***^P < 0.001.

We then explored the effects of *RAB11B* on osteosarcoma cells. In CCK-8 assay, osteosarcoma cells with reduced *RAB11B* displayed a lower proliferation potential than control cells (Figure 9B and Supplementary Figure 6A), suggesting positive role of *RAB11B* in osteosarcoma cells proliferation. Flow cytometer analysis of cell cycle revealed that *RAB11B* increased number of cells in S phase (Figure 9C and Supplementary Figure 6B). Down-regulation of *RAB11B* increased number of apoptotic osteosarcoma cells, indicating an inhibitory role of *RAB11B* in apoptosis of these cells (Figure 9D and Supplementary Figure 7). In addition, we detected the expression of *RAB11B* in the osteosarcoma tissue chips by immunochemistry and examined correlation between *RAB11B* and the clinicopathological characteristics of osteosarcoma. *RAB11B* negatively correlated with the degree of tumor differentiation and age, and positively advanced clinical stage (Figure 9E and Table 2). These results revealed that *RAB11B* promotes osteosarcoma progression.

**Table 2:** The association between clinicopathological characteristics and expression of *RAB11B*

Factors	No of tissues (*n* = 145), *n* (%)	*RAB11B* expression		*P*^b^ value
		Low (*n* = 71), *n* (%)	High (*n* = 74), *n* (%)	
**Age**				
≤ 20	50 (34.5)	18 (36.0)	32 (64.0)	**0.023**
> 20	95 (65.5)	53 (55.8)	42 (44.2)	
**Gender**				
Male	91 (62.8)	49 (53.8)	42 (46.2)	0.127
Female	54 (37.2)	22 (40.7)	32 (59.3)	
**Location**				
Tibia/femur	98 (67.6)	44 (44.9)	54 (55.1)	0.157
Elsewhere	47 (32.4)	27 (57.4)	20 (42.6)	
**Histological type**				
Osteoblastoma	99 (68.3)	43 (43.4)	56 (56.6)	0.051
Else	46 (31.7)	28 (60.9)	18 (39.1)	
**Differentiated degree**				
High/middle	52 (35.9)	36 (69.2)	16 (30.8)	**< 0.001**
Low/undifferentiation	93 (64.1)	35 (37.6)	58 (62.4)	
**TNM**				
T1N0M0	26 (17.9)	10 (38.5)	16 (61.5)	0.237
T2N0M0	119 (82.1)	61 (51.3)	58 (48.7)	
**Clinical stage**				
I	27 (18.6)	19 (70.4)	8 (29.6)	**0.014**
II	118 (81.4)	52 (44.1)	66 (55.9)	

^b^Pearson Chi-Square *P* value.

The expression of RAB11B is reflected by the histochemistry score(H-score).H-score is equal to Σ(pi^*^i), which the ‘pi’ means the percentage of RAB11B-positive cells in the section and the ‘i’ means the tint strength of the cells. And the H-score cut-off based on the median of the H-score of 145 osteosarcoma tissues and classed as low(H-score ≤ 25) or high(H-score > 25) RAB11B expression.

**Figure 10:**
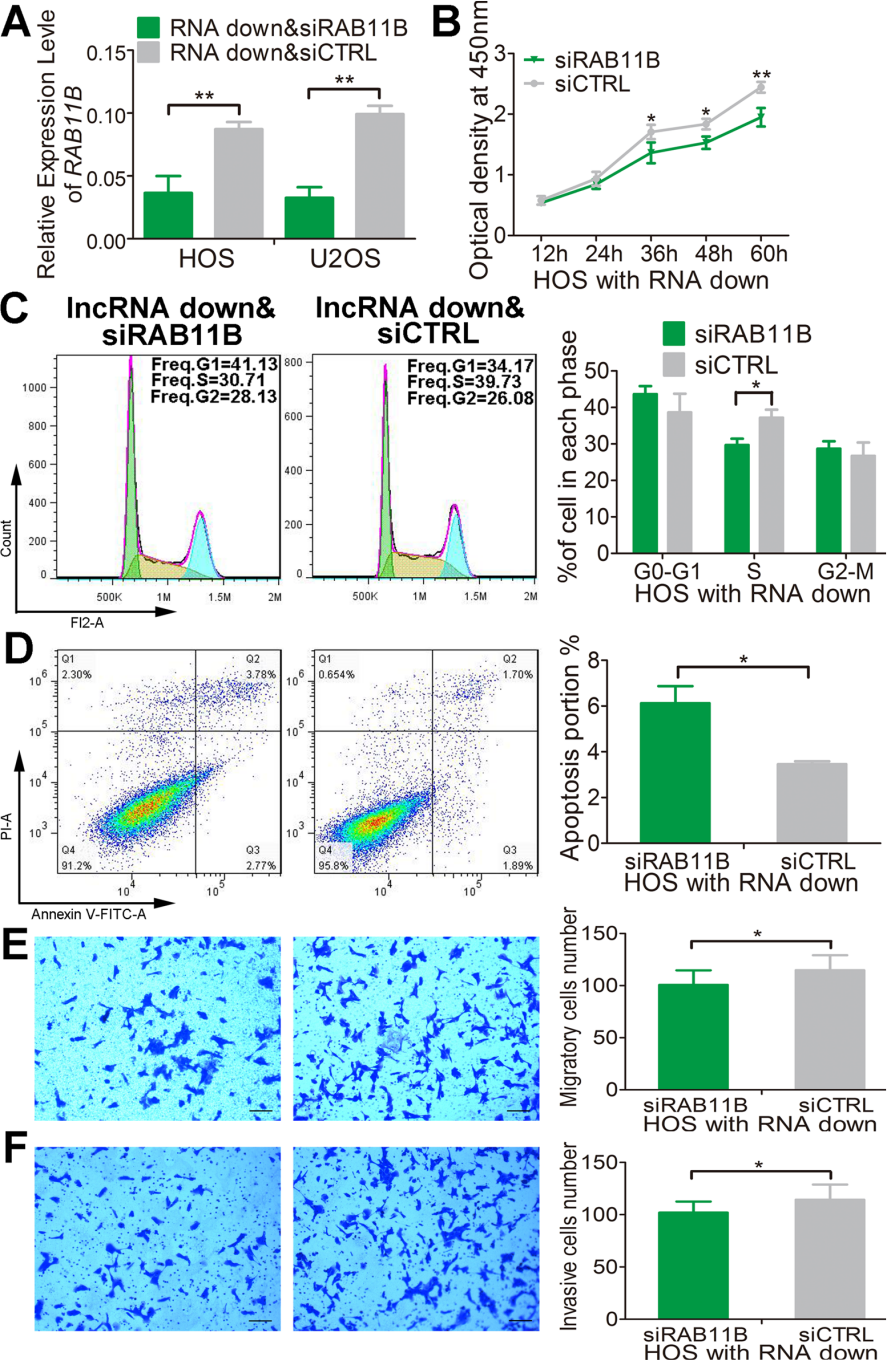
lnc-*RAB11B-AS1* prevents HOS cells proliferation via down-regulating *RAB11B* (**A**) Relative expressions of *RAB11B* were measured by qRT-PCR in HOS and U2OS cells stably down-expressing lnc-*RAB11B-AS1* and transfected with si*RAB11B* or siCTRL. (**B**) HOS cells stably down-expressing lnc-*RAB11B-AS1* was interfered with *RAB11B* expression and subjected to proliferation analysis by CCK-8 assay. (**C**) HOS cells stably down-expressing lnc-*RAB11B-AS1* was interfered with si-*RAB11B* and underwent flow cytometer analysis of cell cycle distribution. (**D**) HOS cells with stable down-regulated lnc-*RAB11B-AS1* was interfered with *RAB11B* expression and subjected to flow cytometer analysis of cell apoptosis. (**E**) HOS cells stably down-expressing lnc-*RAB11B-AS1* was interfered with si-RAB11B and underwent migration assay. (**F**) HOS cells with stable down-regulated lnc-*RAB11B-AS1* was interfered with *RAB11B* expression and subjected to transwell invasion assay. Scale bar, 200 μm. Data was presented as mean ± SD. The results were reproducible in three independent experiments. ^*^P < 0.05, ^**^P < 0.01.

We next define the potential role of *RAB11B* in mediating the effect of lnc-*RAB11B-AS1* in osteosarcoma cells. As depicted above, down-regulation of lnc-*RAB11B-AS1* induced proliferation, migration and invasion and impaired apoptosis of osteosarcoma cells. All of these effects were reversed by *RAB11B* down-regulation (Figure 10 and Supplementary Figures 8–10). These results suggested that lnc-*RAB11B-AS1* exerts its role in osteosarcoma cells via regulating *RAB11B* negatively.

### Aberrant hyper-methylation of the promoter region contributes to decreased lnc-*RAB11B*-AS1 in osteosarcoma

**Figure 11:**
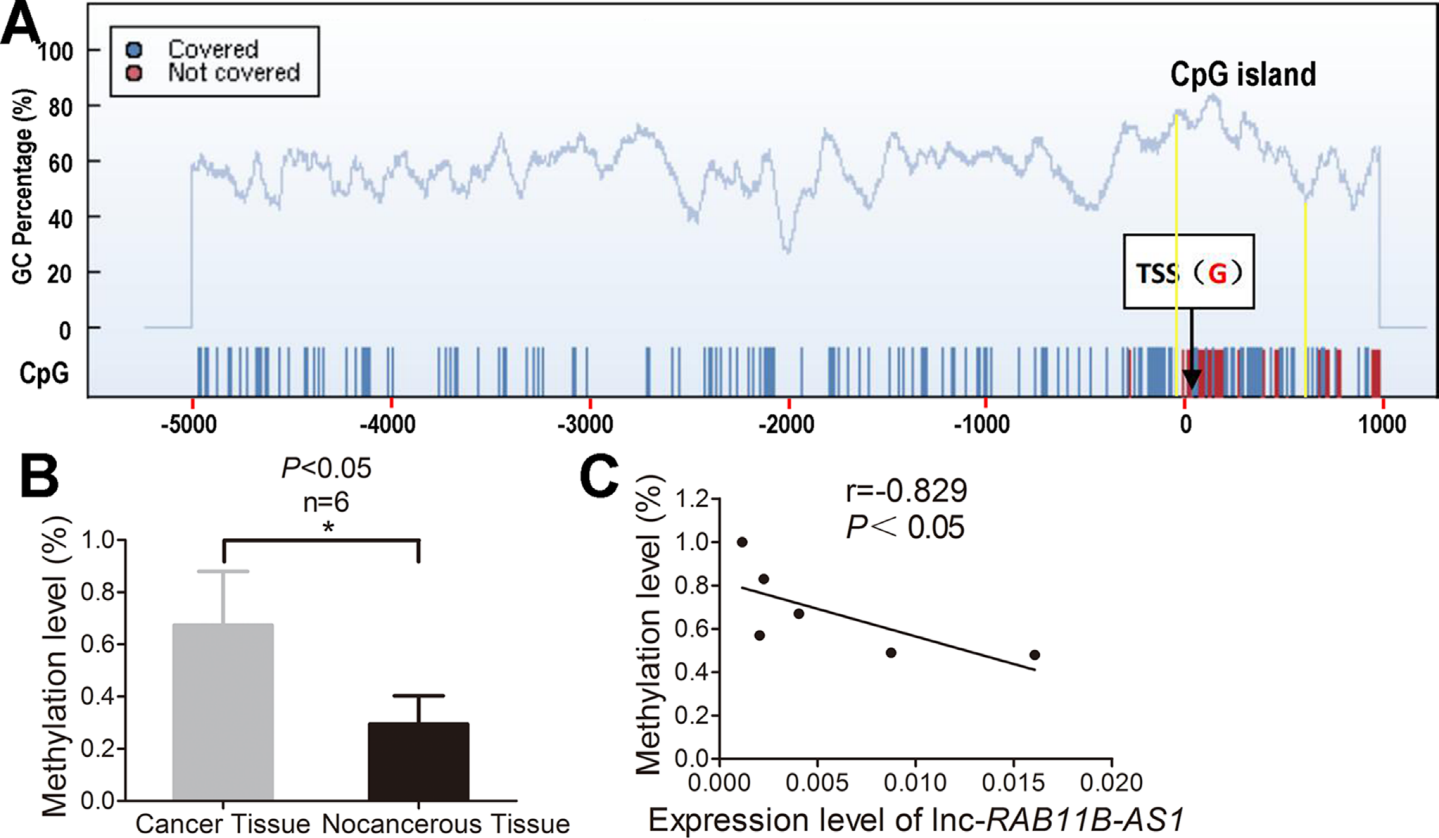
Aberrant hyper-methylation of the promoter region contributes to decresed lnc-*RAB11B-AS1* in osteosarcoma (**A**) One CpG island from 56 bp upstream to 609 bp downsteam of the transcription start site (TSS) in the promoter region of lnc-*RAB11B-AS1* was predicted by the CpG island searcher online software. (**B**) Mean methylation levels of lnc-*RAB11B-AS1* in osteosarcoma and paired non-neoplastic tissues. (**C**) Correlation analysis between mean methylation levels of the promoter region and corresponding lnc-*RAB11B-AS1* expressions. Data was presented as mean ± SD. The results were reproducible in three independent experiments. ^*^P < 0.05.

Emerging evidences now support that DNA methylation is essentially involved in dysregulated lncRNAs expression in a variety of cancer. In light of this notion, we detected the methylation level of CpG island in lnc-*RAB11B-AS1* promoter region to explore the upstream molecular mechanism of down-regulated lnc-*RAB11B-AS1* in osteosarcoma. We found a CpG island from 56 bp upstream to 609 bp downsteam of the transcription start site (TSS) in the promoter region of lnc-*RAB11B-AS1* (Figure 11A). Methylation level of the CpG island was assayed in six osteosarcoma and compared with six paired non-neoplastic tissues after bisulphite conversion of genomic DNA. The mean methylation level of the CpG island in lnc-*RAB11B-AS1* promoter region in osteosarcoma was significantly higher than that in the control samples (Figure 11B). We also investigate the possible associations between methylation level of the CpG island and lnc-*RAB11B-AS1* expression. As shown in Figure 11C, lnc-*RAB11B-AS1* expression correlates negatively with methylation level of the CpG island in osteosarcoma. These data suggest that DNA hyper-methylation in the promoter region of lnc-*RAB11B-AS1* might play an important role in down-regulation of lnc-*RAB11B-AS1* in osteosarcoma.

## DISCUSSION

In recent years, the role of lncRNAs in tumor development and progression attracts more and more investigations. As one kind of the lncRNAs, the antisense lncRNAs get increasing attentions. Previous studies showed critical roles of natural antisense transcripts in many physiological or pathology processes, including tumorigenesis, through regulating sense genes expression [20–23]. Respectable researches have been executed on the antisense lncRNAs in different tumors. For example, the *FEZF1-AS1* is found to be markedly up-regulated in human primary colorectal carcinoma (CRC) and associated with CRC metastasis and poor prognosis [24]. *HIF1A-AS2* was up-regulated in bladder cancer tissues and cells, and *HIF1A-AS2* expression level in bladder cancer tissues is positively associated with advanced clinical pathologic grade and TNM phase [25]. In addition, *EPB41L4A-AS2* inhibits tumor proliferation and is associated with favorable prognoses in breast cancer and other solid tumors [26]. Nonetheless, functions of quite a number of antisense lncRNAs in osteosarcoma remain unclear.

In this study, we found that lnc-*RAB11B-AS1*, a newfound long non-coding RNA, is down-regulated in osteosarcoma tissues as compared with pair-matched noncancerous tissues. Moreover, we identified the role of lnc-*RAB11B-AS1* in osteosarcoma cells. Our results demonstrated that lnc-*RAB11B-AS1* inhibits osteosarcoma cells proliferation, cell cycle progression, invasion and migration *in vitro* and prevents tumor growth *in vivo*, indicating lnc-*RAB11B-AS1* as an anti-oncogene in osteosarcoma. Mechanistically, lnc-*RAB11B-AS1* exerts these effects by binding to *RAB11B* 5’UTR and reducing *RAB11B* expression. The correlation between *RAB11B* and *RAB11B-AS1* in osteosarcoma tissues supports the role of *RAB11B* in mediating the effects of *RAB11B-AS1*. In addition, luciferase reporter assay illustrated the interaction between *RAB11B-AS1* and the *RAB11B*. Most importantly, all the effects of lnc-*RAB11B-AS1* were abrogated by *RAB11B* down-regulation, which validated that lnc-*RAB11B-AS1* exerts its function in osteosarcoma via *RAB11B*.

Recently, epigenetic silencing of tumor-suppressor lncRNA by aberrant DNA hyper-methylation attracts increasing attention in a variety of cancer [27–29]. To explore the epigenetic mechanism by which lnc-*RAB11B-AS1* is regulated in osteosarcoma, we assessed the DNA methylation status of promoter region of lnc-*RAB11B-AS1* in six paired osteosarcoma and adjacent normal samples. As expected, lnc-*RAB11B-AS1* expression correlates negatively with DNA methylation of its promoter region. We speculate that hyper-methylation of the promoter region might abrogate lnc-*RAB11B-AS1* expression, and inhibition of methylation in lnc-*RAB11B-AS1* might be beneficial for treatment of osteosarcoma.

A big challenge in treatment of osteosarcoma is its resistance to the standard chemotherapy. Cisplatin is one of the most important chemotherapeutic drugs in conventional chemotherapy for osteosarcoma. However, multiple and complex genetic and epigenetic factors affect the sensitivity of osteosarcoma to cisplatin [30]. In present study, we detected the effect of lnc-*RAB11B-AS1* on cisplatin treatment in osteosarcoma. We found that lnc-*RAB11B-AS1* increased the sensitivity of osteosarcoma cells to cisplatin obviously. Since lnc-*RAB11B-AS1* was revealed to inhibit proliferation, migration and invasion and promote the apoptosis of osteosarcoma cells, lnc-*RAB11B-AS1* is a potential therapeutic target for osteosarcoma.

In conclusion, the present study revealed lnc-*RAB11B-AS1* as an essential molecular marker for osteosarcoma development and progression and a potential therapeutic target for osteosarcoma.

## MATERIALS AND METHODS

### Ethics statement

Each participant was scheduled for an interview to collect individual information and to obtain one pair of osteosarcoma samples and adjacent tissues under his or her informed consent. The study was approved by the institutional review boards of the Third Affiliated Hospital of Southern Medical University.

### Sample collection

Patients with osteosarcoma, who underwent surgery at the Third Affiliated Hospital of Southern Medical University between 2015 and 2016, were retrospectively selected for the present study. A total of 24 pairs of osteosarcoma samples and corresponding non-neoplastic tissues were used. In addition, clinical information and characteristics of patients were also collected.

### Cell lines and tissue chip

The U2OS, Sao-2, MG-63 and MNNG/HOS Cl#5[R-1059-D] human osteosarcoma cell lines were purchased from the Cellcook Biotech Company (Guangzhou, China). The OS cell lines were cultured at 37°C in 90% humidity and 5% carbon dioxide (CO2) in MEM medium (Gibco, life technologies, California, USA) with 10% fetal bovine serum (FBS) and penicillin (100 UI/mL)/streptomycin (100 mg/mL) in SANYO AUTOMATIC CO_2_ INCUBATOR (SANYO Electric Co., Ltd., Japan). The hFOB1.19, human osteoblast transfected by SV40, was purchased from the Yuesui Biotech Company (Guangzhou, China) and it was cultured at 34°C in DMEM medium (Gibco, life technologies, California, USA). The osteosarcoma tissue chips which include 145 cases of osteosarcoma tissue were purchased from Alenabio Company (Xi’an, China).

### Quantitative real-time polymerase chain reaction

All the osteosarcoma tumor tissues and matched adjacent normal tissues were identified using quantitative real-time polymerase chain reaction (qRT-PCR). We also identified the expression level of the related lnc-*RAB11B-AS1* and the target mRNA *RAB11B*. All primers are listed in the Supplementary Table 1.

### Subcellular fractionation

In order to determine the cellular localization of lnc-*RAB11B-AS1*, cytosolic and nuclear fractions were collected according to the manufacturer's instructions for nuclear/cytoplasmic isolation kit (Biovision, San Francisco, CA).

### Plasmids construction, lentivirus package and transduction

The full-length complementary DNA of human lnc-*RAB11B-AS1* and small hairpin RNA (shRNA) are both synthesized by iGeneBio (Guangzhou, China). The full-length complementary DNA of human lnc-*RAB11B-AS1* was cloned into the lentiviral expression vector pEZ-Lv206 for over-expression and small hairpin RNA (shRNA) of this human lnc-*RAB11B-AS1* was cloned into vector psi-LVRH1MP for gene silencing. The resulting construct (pEZ-Lv206-lnc-*RAB11B-AS1* and psi-LVRH1MP- lnc-*RAB11B-AS1*) was verified by sequencing. And the control groups are their respective empty vector. All sequences are listed in the Supplementary Material.

### Cell viability and proliferation assay

Cell viability of cells was measured by using Cell Counting Kit-8 system (CCK-8, Engreen Biosystem Co. Ltd., China) according to the manufacturer's instructions. Proliferation rates were determined at 12, 24, 36, 48 and 60 hours. Cells were incubated in 10% CCK-8 diluted in normal culture media at 37°C until the visual color conversion occurred. The absorbance of each well was measured with a microplate reader set at 450 nM.

### Cell migration assay and invasive assay

Cell migration assay was appraised by Corning transwell insert chambers (Costar, Corning Incorporated, NY). Cells were grown to near confluence in a 5 × 5 culture vessel and then placed in serum-free MEM (Gibco, life technologies, California, USA) for 24 hours. Cells were trypsinized and re-suspended in serum-free MEM with 0.1% BSA. We planted 2 × 10^4^ cells in the chamber, which was put in a 24-well plate contain MEM with 20% fetal bovine serum (FBS, Gibco, life technologies, California, USA). After 12 hours culture, non-migrating cell in the top of the chambers were removers by swabbing. Migrated cells were counted manually in 10 random fields and their numbers were averaged. Cells invasive assay was conducted exactly the same, but with an 8-μm Corning Matrigel invasive chamber (Corning Incorporated, NY., USA).

### Flow cytometry analysis of cell cycle and apoptosis

In the cell-cycle analysis, cells were labeled with propidium iodide (PI) (sigma, St. Louis, MO) and analyzed by flow cytometry. For apoptosis analysis, Annexin V-fluorescein isothiocyanate (FITC)/PI straining also was performed by using flow cytometry according to the manufacturer's guidelines (MULTISCIENCES, Hangzhou, China).

### Luciferase reporter assay

pGL3-Promoter-*RAB11B* vector and pGL3-Promoter-control vector was co-transfected with lnc-*RAB11B-AS1* up-regulation or down-regulation using Lipofectamine 3000 (Invitrogen) into osteosarcoma cells. The relative luciferase activity was normalized to Renilla luciferase activity 48 h after transfection. All sequences are listed in the Supplementary Table 2.

### Western blot

Identical quantities of proteins were separated by sodium dodecyl sulfate-polyacrylamide gel electrophoresis (SDS-PAGE), transferred onto PVDF membranes, and subjected to western blot analysis by using anti-*RAB11B* (Abcam, USA), following the manufacturer's instructions.

### Transient transfection

The siRNA sequence of *RAB11B* was synthesized by the RIBOBIO CO., LTD (Guangzhou, China). All sequences are listed in the Supplementary Material. The osteosarcoma cells were transfected with si-RAB11B or si-CTRL using Lipofectamine 3000 reagent and Opti-MEM I Reduced Serum Medium (Gibco, life technologies, California, USA) in accordance with the manufacturer's instructions, respectively.

### Susceptibility testing

We adjusted inoculation quantity to 90% of each well base area in 96-well plates and then added a concentration gradient of cisplatin (0 μg/ml, 1 μg/ml, 2 μg/ml, 4 μg/ml, 8 μg/ml, 16 μg/ml, 32 μg/ml). After 24 hours, cells were incubated in 10% CCK-8 diluted in normal culture media at 37°C until the visual color conversion occurred. The absorbance of each well was measured with a microplate reader set at 450 nM.

### Orthotopic xenotransplantation

Male BALB/c nude mice aged 5 weeks were purchased from Southern Medical University Animal Center (Guangzhou, China) and maintained under specific pathogen free (SPF) condition in the animal care facility at Southern Medical University. A total of 2 × 10^6^ HOS cells with knockdown, overexpression or controls of lnc-*RAB11B-AS1* were suspended in 0.2 ml normal saline and then subcutaneously injected into the back of each mouse. The length (L, mm) and width (W, mm) of tumors were measured every 3 days starting the 7^th^ day after inoculation. Tumor volume was calculated using the formula V = W^2^ x L x 0.5. 19 days later, the mice were euthanized, necropsies were performed, and tumors were weighed. All procedures were monitored in accordance with the ethical standards and the care of animal and licensing guidelines, under the protocol approved by the Committee on Animal Welfare of Southern Medical University.

### Immunohistochemistry

Tissues were blocked by 10% formalin and embedded in paraffin. The paraffin-embedded tissues were cut into 4 μm-thick section and then the sections were de-waxed in xylene and rehydrated in a graded alcohol series. Antigen retrieval was conducted. H_2_O_2_ (3%) was used to suppress endogenous peroxidase activity. Polyclonal rabbit antibody at 1:100 dilution against Ki-67 (Abcam, USA) was added and incubated at 4°C overnight. After washing, sections were incubated with anti-rabbit secondary antibody at 1:100 dilution for 1 h at 37°C. Staining was detected by diaminobenzidine (DAB). Sections were counterstained with hematoxylin.

### TUNEL staining

To detect apoptotic cells on the same sections, TUNEL staining was performed using DeadEnd™ Fluorometric TUNEL System (G3250, Promega, America) following the manufacturer's protocol. The samples were mounted in ProLong Gold antifade mountant with DAPI (Invitrogen, America). Images were obtained using an AXIO-Scope.A1 (ZEISS, Germany). These counts were averaged to obtain the number of apoptotic cells per specimen.

### CpG island prediction

The lnc-*RAB11B-AS1* sequence (5000 bp upstream of the TSS to 1000 bp downsteam of the TSS) was download from NCBI (https://www.ncbi.nlm.nih.gov/) and predicted the distribution of CpG islands using the CpG island searcher online software (http://www.ebi.ac.uk/Tools/seqstats/emboss_cpgplot/) with the parameters set as lower limits: (%GC>50, ObsCpG/ExpCpG>0.6, length>200).

### DNA methylation sequencing by MassArray

The pair of primers spanning predicted CpG islands of lnc-*RAB11B-AS1* were designed using the EpiDesigner software (http://www.epidesigner.com). Primer sequences were: forward primer: 5′-aggaagagagAATTTTGGGA AAGTTTTATTTTTTG-3′; reverse primer: 5′- cagtaatacgactcactatagggagaaggct CCTCAAAACACTACTTCCATCTCTA-3’. Genomic DNA was extracted from 6 osteosarcoma specimens and their adjacent normal tissues using the genomic DNA extraction kit (BioTeKe Corpration, Beijing, China). The genomic DNA from each sample was treated with sodium bisulfite using an EZ DNA methylation kit (Zymo Research, Orange, CA). Quantitative methylation analyses of the CpG sites were performed using the Sequenom MassARRAY platform (CapitalBio, Beijing, China). The spectral methylation ratios were analyzed with EpiTyper software version 1.0 (Sequenom, San Diego, CA, USA).

### Statistics

All results are presented as the mean ± S.D. Difference between groups were assessed by student's *t*-test (two tailed). The relationship between lnc-*RAB11B-AS1* and *RAB11B* expression was analyzed by Pearson's correlation. Others comparisons were determined by χ^2^ test or Analysis of Variance (ANOVA). The SPSS 18.0 software package (SPSS, Chicago, IL, USA) was used for statistical analysis. The *P* value less than 0.05 was considered to be significant.

## SUPPLEMENTARY MATERIALS FIGURES AND TABLES


