# Supplementary material for: Long non-coding RNA RAB11B-AS1 prevents osteosarcoma development and progression via its natural antisense transcript RAB11B
Source: Oncotarget. 2018 Jan 13;9(42):26770–86. doi: 10.18632/oncotarget.24247 (PMC6003561; doi:10.18632/oncotarget.24247)
Supplement: Supplementary file 1 [file oncotarget-09-26770-s001.pdf]

Long non-coding RNA *RAB11B-AS1* prevents osteosarcoma development and progression via its natural antisense transcript *RAB11B*

SUPPLEMENTARY MATERIALS

Supplementary Material: the sequences which are related to the study

lnc-RAB11B-AS1 over-expression

5′-GCCCCGGCGCGTCCTAGGTCCCCCAG
GTCTCTCCGGGCCTCCGGCTCCGTATAGC
CGCGCATCCTAGCCCGAAGCTGATGCTGCCC
GGTCGGCTCCGGCCTTCCCGGCTGCCT
GACCCGCCGGGCCCCAAGCCCCGGGCTC
CGGCCTGGGCCCAGCGGCCTGGAGCGCG
GCCTTGGCCCGTCTGCCCGCCGCCTCGCCGC
CACGACTTCGCCCGTCTGTGCCCCACCGGCCG
CACCTTTGAATAGGTAGTCGTACTCGTCGTCCC
GGGTCCCCATTGTCCTGGCGCTTCCGGCGGGATC
GGCGACTCCGCAGCCCCACCACAAACACCC
GACGGGGGCGGAGCCGGCGCCGCGCAGAGCG
GCGGTTGAATGGCCTATCAGCGGCGAGTGGAG
TAGCGACGGGCACCCAGCGAAGCCAATCAGA
GATGGAAGTAGTGCTCTGAGGGTGGGCGCC
GCTTGGTACCACCCTCCTCGCCCTCGGTGTCCTG
GAGAAAGGCGGAAGGAATGCGGACCTTTTT
GAAGTGACGGACGCGCCAGCCTATCAGGGGC
GAGCTCAAGAGGGCGGGGCGGAAGACTG
CAGGAATGAAATGGATTGACAGACCAAATA
ACTAATGAGAGGCTTGATTGAGAACCTACCC
GACTATCAGAGGACCTGTCCGGGAAGAGA
AATGGGGCTACGTCCAGACAGAATCTCGCTCT
GTTGCCTGGGCTGGAGTGCAGTGGCACTG
TATCCGGAATTGGGGGGTTCTTGGTCTCACT
GACTTCAAGAATGAAGCCATGGACCCTTGCGAGA
TGGGGGTTCCACCATGTTGGCCCGGCTGGTCTC
GAACTGCTGACCTCAAGTGACCTGCCTGTCTCG
GCCTCACAAATGGCTGGGATTACAGGCACGAGA
CACCGTGCCTGGCCCTGGGAACATGTTTACATG
GACTTTGTTCACTTTTTAAAACAAACTGAGGG
TAACTTACTAGATCTGTGAACTAAAGAAAGAGA
AAAAAAAGAAAGAAAACAAACAAGAA
CAAAGAA-3′

lnc-RAB11B-AS1 shRNA:

5′-GCGGAAGACTGCAGGAATGAA-3′

RAB11B promoter:

5′-CCTGGGCAACGACAGCAAAACTCCGTCT
CAAAAAAAAAAAAAAAAAACTGTCATAATTAT
TATTTCACTGGGTACATGTGTATCAAAACACAC
GCTGTACACCCTAAATATATAAAACAAACA
CATACTGTATTGTCAGGTAGTGATAAATCCTAT
CACTACCTGACAATACAGCAGTATGTGTTT
TATTTTCTATATTACCTATTTTTATATTTTACCTAT
GTGGCAAAATATAGGACAAAAGAGGGGTAGTG
GTAGGAGGTCCTGTTGGAGAGCATTGGTATCCAG
TAAGGATTTAAAAGTATTTATTTAACAAAGAATAG
CAGAAAGTGGACGGTTGATTCACACTTGGTG
CAATCTCGGCTCACTGCAACCTCCACCTCCCAG
GTTCAAGCGATTCTCCTGCCTCAACCTCCTGAG
TAGCTGGGTTTACAGGCACCAGCGACCAC
GCCCGACTAATTTTTGTATTTTTAGTAGAAAC
GGGGTTTCACCATGTTGGTCAGGCTGGTCTC
GAACTCCTGACCTCGTGATCCGCCAGCCTCG
GCCTCCCAAAGTCCTGGAATTACAGGCGTGAGC
CACCGCGCCTGGCTGATCTGGTTATTTTCAAAA
GATTCCATGAGACGGGTTTCTTCAGTTTACAGAG
GAGGAAACTGAGGCACAGAAGGATAACCCAGT
GAGTCCCAATATAGAGATGGAGACTGCAAC
CGAGAAAGGGGACCCAGCGCTGAGGTCCCCG
GAAATGGTCATTTATGGGCTTGGGGGGCGAC
CAGGGCAGCGCGCGCTGACCTATGACGTCATC
GGGACGTTAAGCATCGTAGCATTACCTGGACG
TAGCCCCATTTCTCTTCCCGGACAGGTCCTCT
GATAGTCGGGTAGGTTCTCAATCAAGCCTCT
CATTAGTTATTTGGTCTGTCAATCCATTTCATTCCT
GCAGTCTTCCGCCCCGCCCTCTTGAGCTCGCCCCT
GATAGGCTGGCGCGTCCGTCACTTCAAAAAGGTC
CGCATTCCTTCCGCCTTTCTCCAGGACAC
CGAGGGCGAGGAGGGTGGTACCAAGCGGC
GCCCACCCTCAGAGCACTACTTCCATCTCTGATTG
GCTTCGCTGGGTGCCCGTCGCTACTCCACTCGC
CGCTGATAGGCCATTCAACCGCCGCTCTGCGCG
GCGCCGGCTCCGCCCCCGTCGGGTGTTTGTG
GTGGGGCTGCGGAGTCGCCG
ATCCCGCCGGAAGCGCCAGGACAATGGGGAC
CCGGGACGACGAGTACGACTACCTATTCAAAG-3′

RAB11B siRNA:

5′-GCAACATCGTCATCATGCT-3′

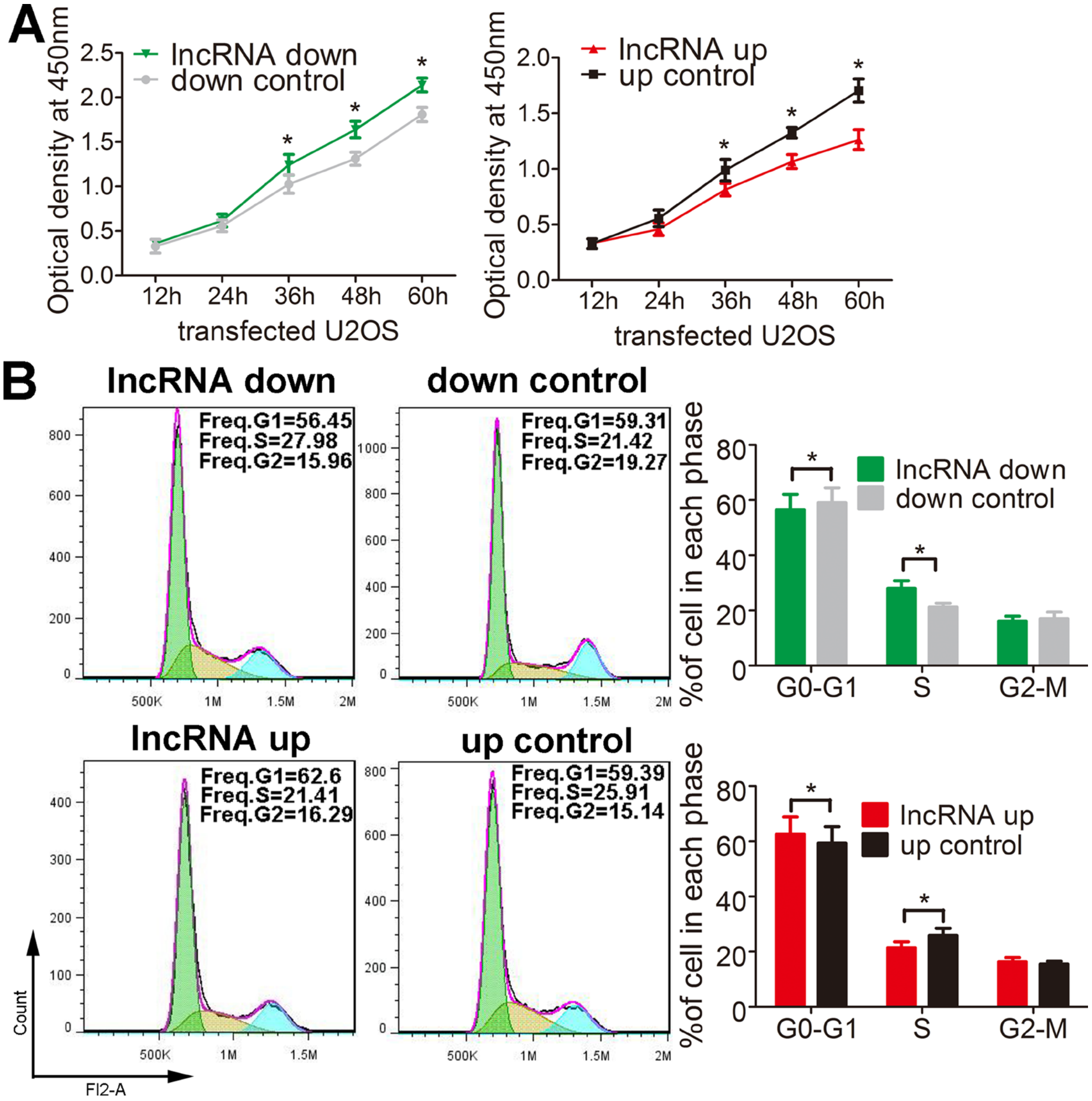

Supplementary Figure 1: lnc-*RAB11B-AS1* inhibits U2OS cells proliferation. (**A**) Proliferation of U2OS cells with up-regulated or down-regulated lnc-*RAB11B-AS1* was determined by CCK-8 assay. (**B**) Flow cytometer analysis of the cell cycle distribution of U2OS cells with up-regulated or down-regulated lnc-*RAB11B-AS1*. Data was presented as mean ± SD. The results were reproducible in three independent experiments. $^{*}P < 0.05$.

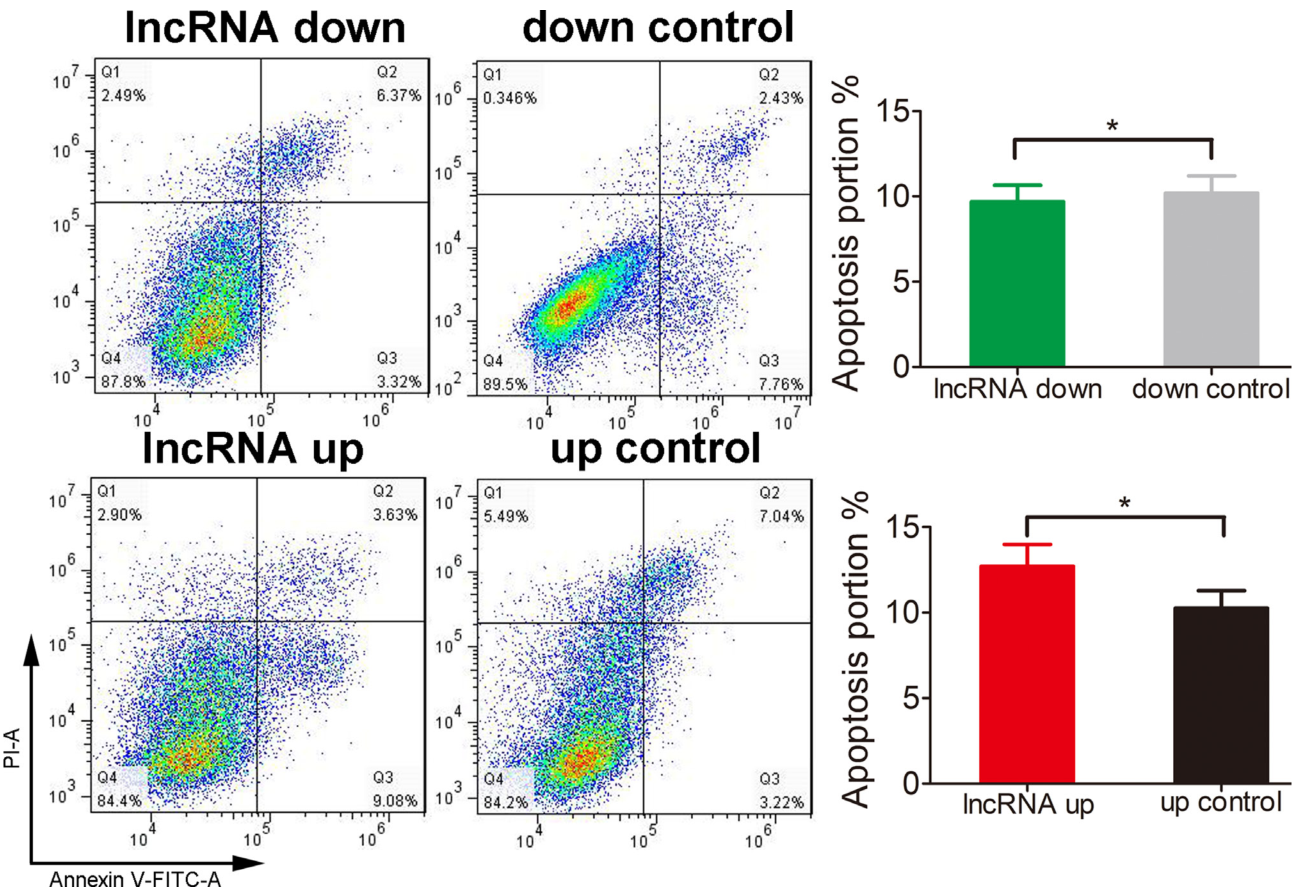

Supplementary Figure 2: lnc-*RAB11B-AS1* promotes U2OS cells apoptosis. Annexin V-FITC/PI apoptosis assay of U2OS cells with up-regulated or down-regulated lnc-RAB11B-AS1. Data was presented as mean ± SD. The results were reproducible in three independent experiments. $^{*}P < 0.05$.

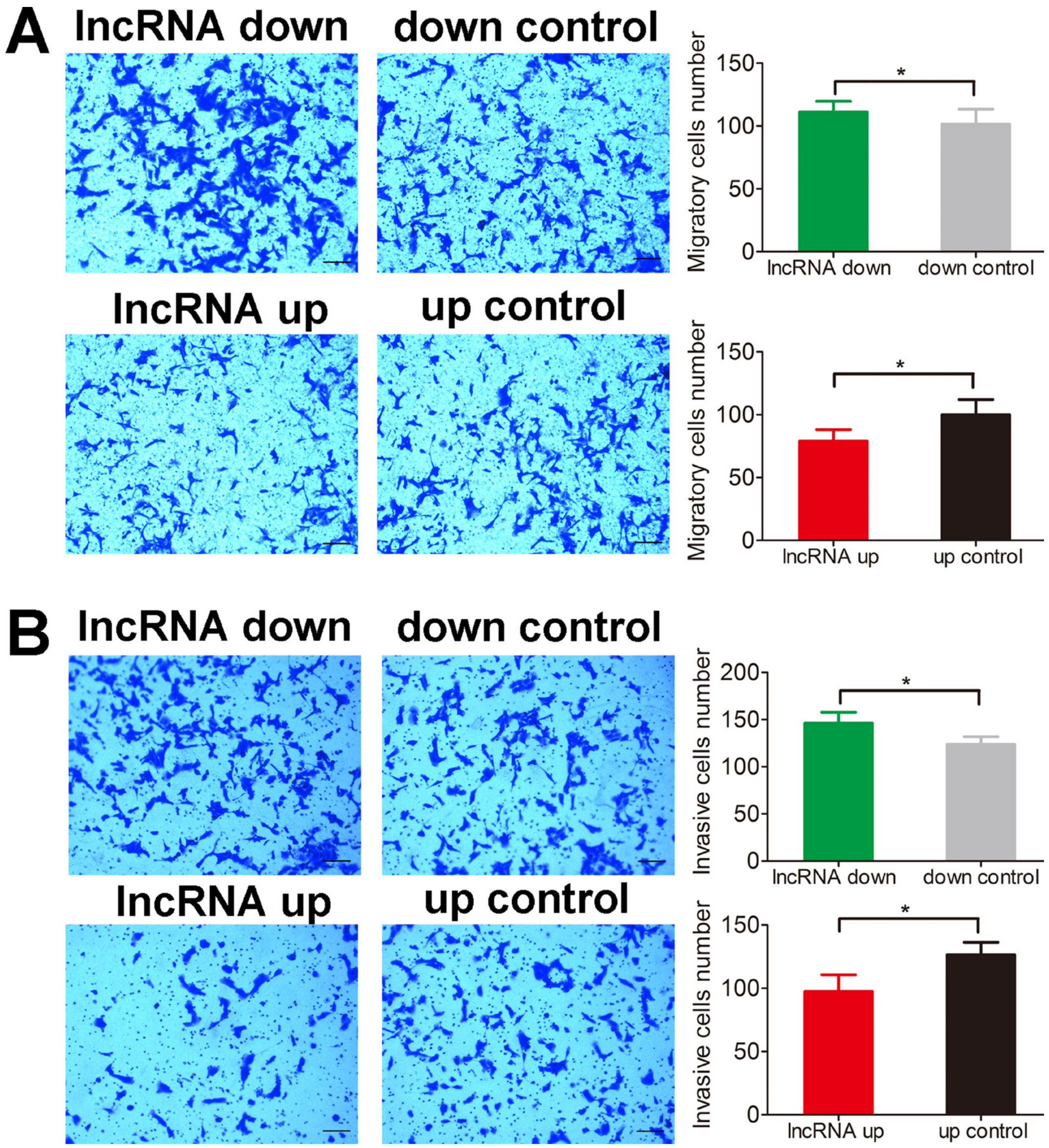

Supplementary Figure 3: lnc-*RAB11B-AS1* impaires migration and invasion of U2OS cells. (**A**) Migration assay of U2OS cells with up-regulated or down-regulated lnc-*RAB11B-AS1*. (**B**) Transwell invasion assay of U2OS cells with up-regulated or down-regulated lnc-*RAB11B-AS1*. Migration and invasion capacities of osteosarcoma cells were measured by transwell chamber assay, and the photographs were randomly selected and taken at × 100 field. Scale bar, 200 μm. Data was presented as mean ± SD. The results were reproducible in three independent experiments. $^{*}P < 0.05$.

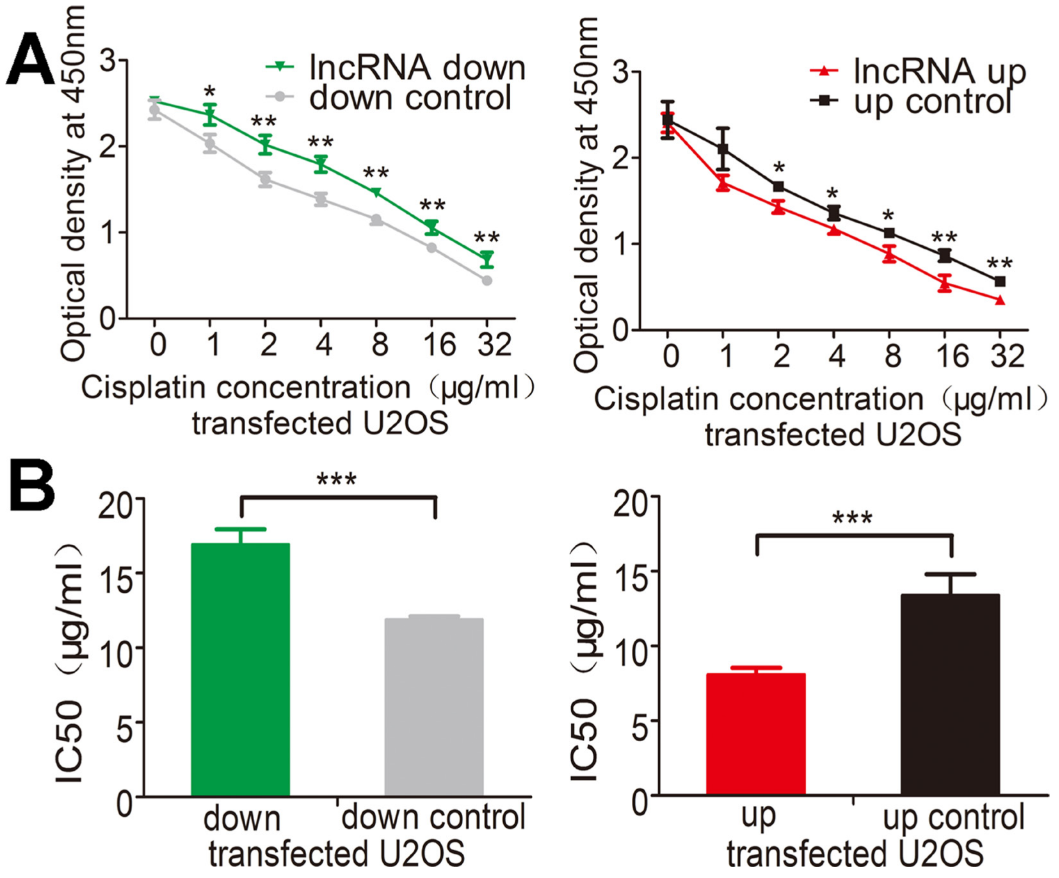

Supplementary Figure 4: lnc-*RAB11B-AS1* elevates sensitivity of U2OS cells to cisplatin. (**A**) U2OS cell with down-regulated or up-regulated *RAB11B-AS1* were added with increasing concentration of cisplatin and subjected to CCK-8 assay. (**B**) U2OS cells with lnc-*RAB11B-AS1* up-regulation were more sensitive to cisplatin (IC50 = 21.15μg/ml for lnc-RAB11B-AS1 down-regulation group and IC50 = 10.25μg/ml for lnc-*RAB11B-AS1* up-regulation group). Data was presented as mean ± SD. The results were reproducible in three independent experiments. $^{*}P < 0.05$, $^{**}P < 0.01$, *** $P < 0.001$.

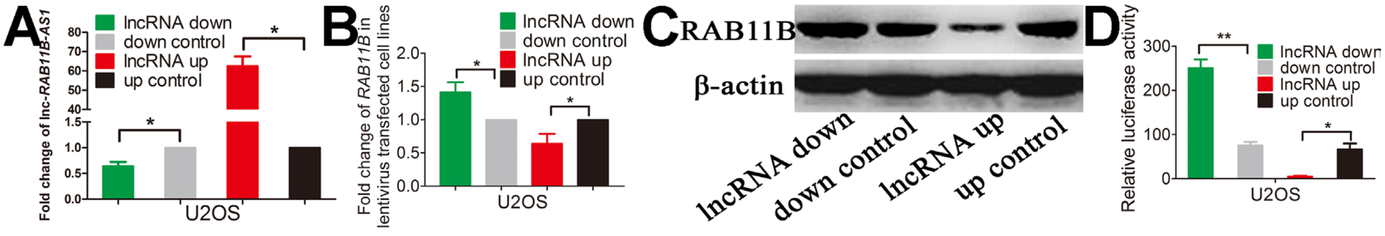

Supplementary Figure 5: lnc-*RAB11B-AS1* correlates negatively with its sense-cognate gene *RAB11B* in U2OS cells. (**A**) qRT-PCR analysis of *RAB11B-AS1* in lentivirus transfected U2OS cells. (**B**) qRT-PCR analysis of *RAB11B* in U2OS cells with down-regulated or up-regulated *RAB11B-AS1*. (**C**) Western blot analysis of *RAB11B* in U2OS cells with down-regulated or up-regulated *RAB11B-AS1*. (**D**) The relative luciferase activity of pGL3-Promoter-*RAB11B* was markedly increased in U2OS cells with down-regulated lnc-*RAB11B-AS1* and was reduced in cells with up-regulated lnc-*RAB11B-AS1*. Data was presented as mean ± SD. The results were reproducible in three independent experiments. $^{*}P < 0.05$, $^{**}P < 0.01$.

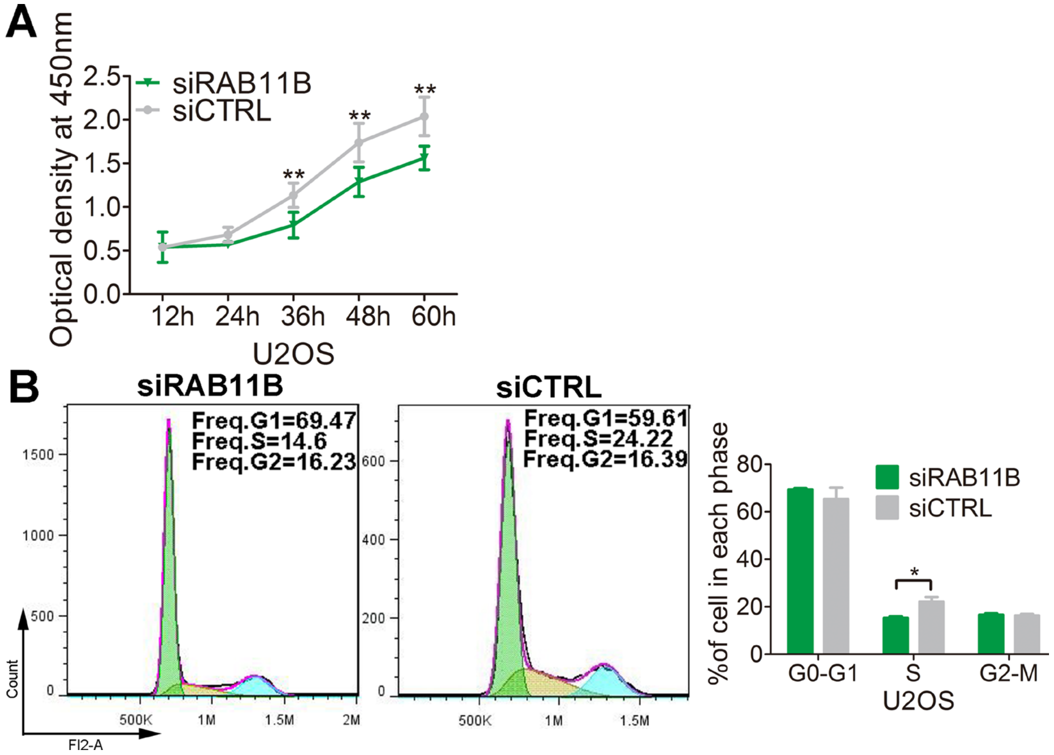

Supplementary Figure 6: *RAB11B* promotes proliferation of U2OS cell. (**A**) Proliferation of U2OS cells with disrupted *RAB11B* or not was determined by CCK-8 assay. (**B**) Flow cytometer analysis of cell cycle distribution in U2OS cells with RAB11B down-regulated or not. Data was presented as mean ± SD. The results were reproducible in three independent experiments.$^{*}P < 0.05$, $^{**}P < 0.01$.

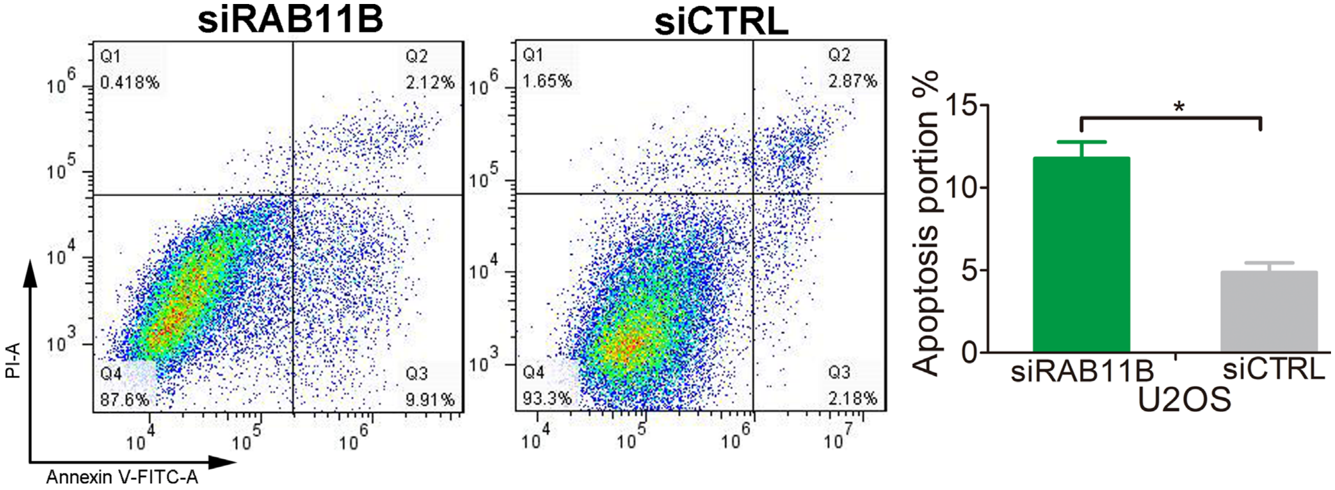

Supplementary Figure 7: *RAB11B* inhibites apoptosis of U2OS cells. Flow cytometer analysis of apoptosis in U2OS cells with *RAB11B* down-regulated or not. Data was presented as mean ± SD. The results were reproducible in three independent experiments. $^{*}P < 0.05$.

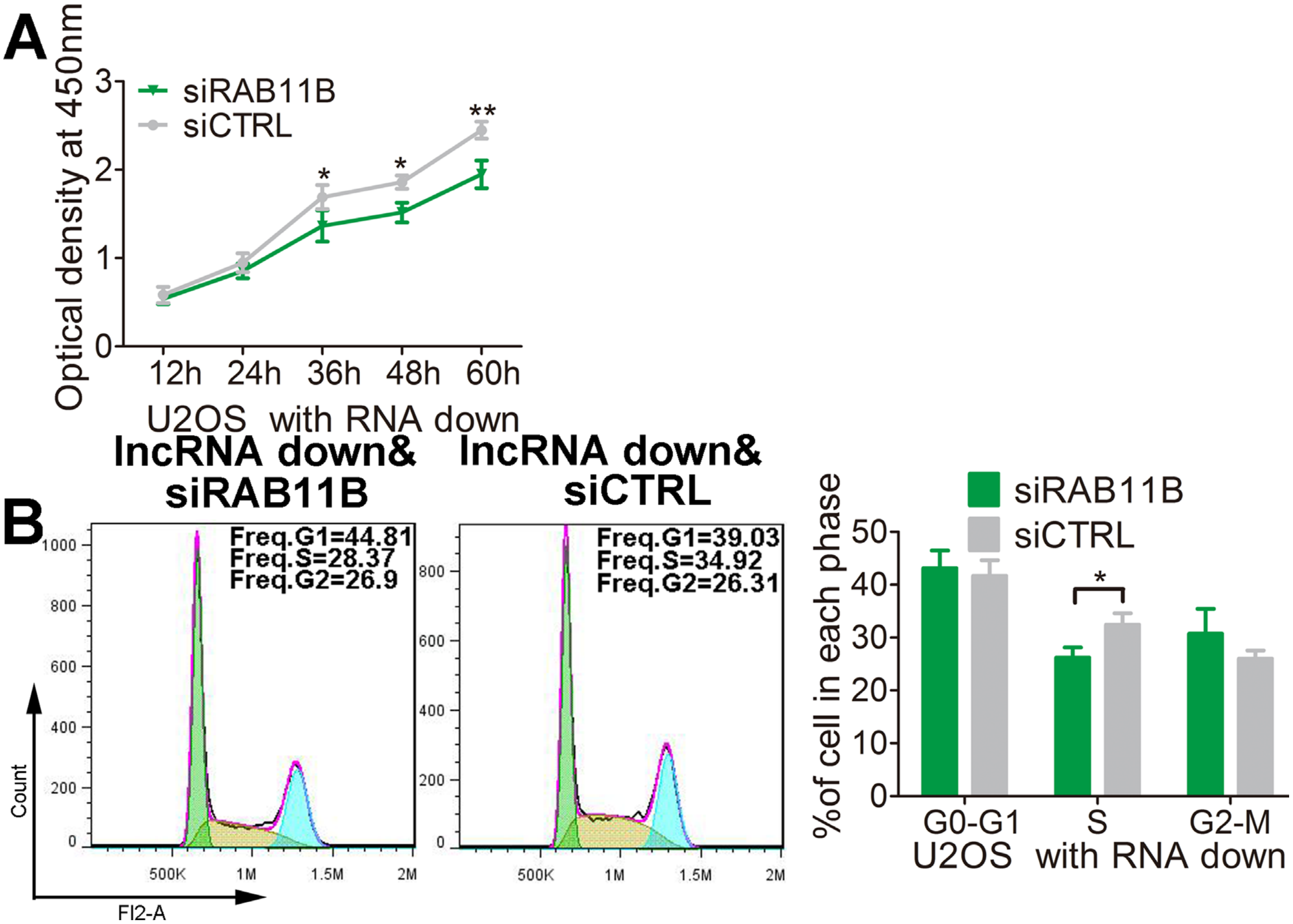

Supplementary Figure 8: lnc-*RAB11B-AS1* preventes U2OS cells proliferation via down-regulating *RAB11*B. (**A**) U2OS cells stably down-expressing lnc-*RAB11B-AS1* was interfered with *RAB11B* expression and subjected to proliferation analysis by CCK-8 assay. (**B**) U2OS cells stably down-expressing lnc-*RAB11B-AS1* was interfered with si-RAB11B and underwent flow cytometer analysis of cell cycle distribution. Data was presented as mean ± SD. The results were reproducible in three independent experiments. $^{*}P < 0.05$, $^{**}P < 0.01$.

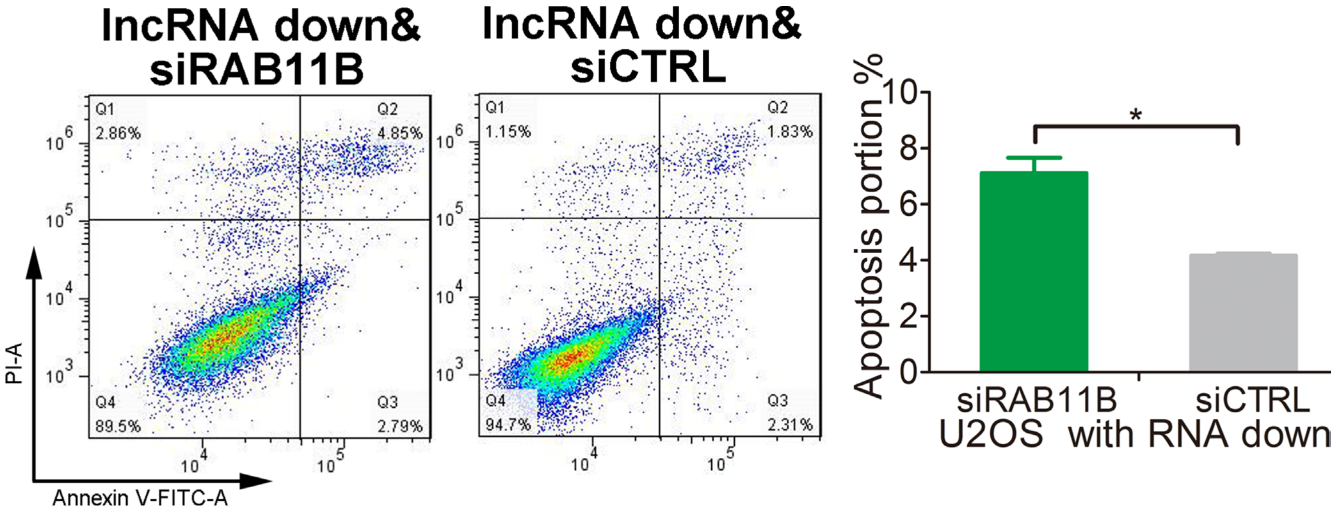

Supplementary Figure 9: lnc-*RAB11B-AS1* promotes U2OS cells apoptosis via down-regulating *RAB11B*. U2OS cells with stable down-regulated lnc-*RAB11B-AS1* was interfered with *RAB11B* and subjected to flow cytometer analysis of cell apoptosis. Data was presented as mean ± SD. The results were reproducible in three independent experiments. $^{*}P < 0.05$.

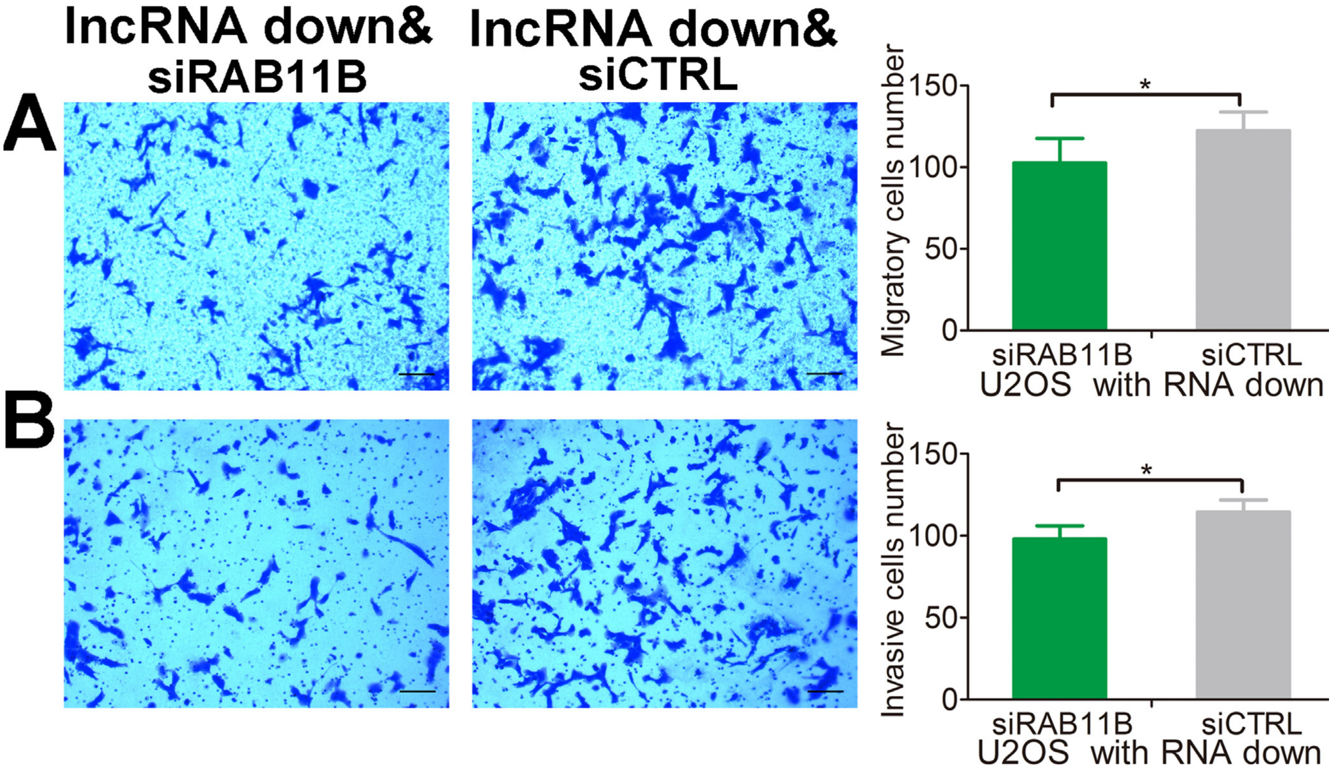

Supplementary Figure 10: lnc-*RAB11B-AS1* prevents U2OS cells migration and invasion via down-regulating *RAB11B*. (**A**) U2OS cells stably down-expressing lnc-*RAB11B-AS1* was interfered with si-*RAB11B* and underwent migration assay (**B**) U2OS cells with stable down-regulated lnc-*RAB11B-AS1* was interfered with *RAB11B* expression and subjected to transwell invasion assay. Migration and invasion capacities of osteosarcoma cells were measured by transwell chamber assay, and the photographs were randomly selected and taken at × 100 field. Scale bar, 200 µm. Data was presented as mean ± SD. The results were reproducible in three independent experiments. $^{*}P < 0.05$.

Supplementary Table 1: The primers

Gene	Forward primer	Reverse primer
**lnc-*RAB11B-AS1***	GGAACATGTTTACATGGACTTTGT	TCTTTGTTCTTGTTTGTTTTCTTTCT
**RAB11B**	CGTACTACCGTGGTGCAGTG	ATGACGATGTTGCTGTCTGC
**β-actin**	GGCGGCACCACCATGTACCCT	AGGGGCCGGACTCGTCATACT
**U-6**	CTCGCTTCGGCAGCACA	AACGCTTCACGAATTTGCGT
